# Supplementary material for: Deep learning enabled integration of tumor microenvironment microbial profiles and host gene expressions for interpretable survival subtyping in diverse types of cancers
Source: mSystems. 2024 Nov 20;9(12):e01395-24. doi: 10.1128/msystems.01395-24 (PMC11651096; doi:10.1128/msystems.01395-24)
Supplement: Supplemental material — Supplemental figures and tables. [file msystems.01395-24-s0001.docx]

**
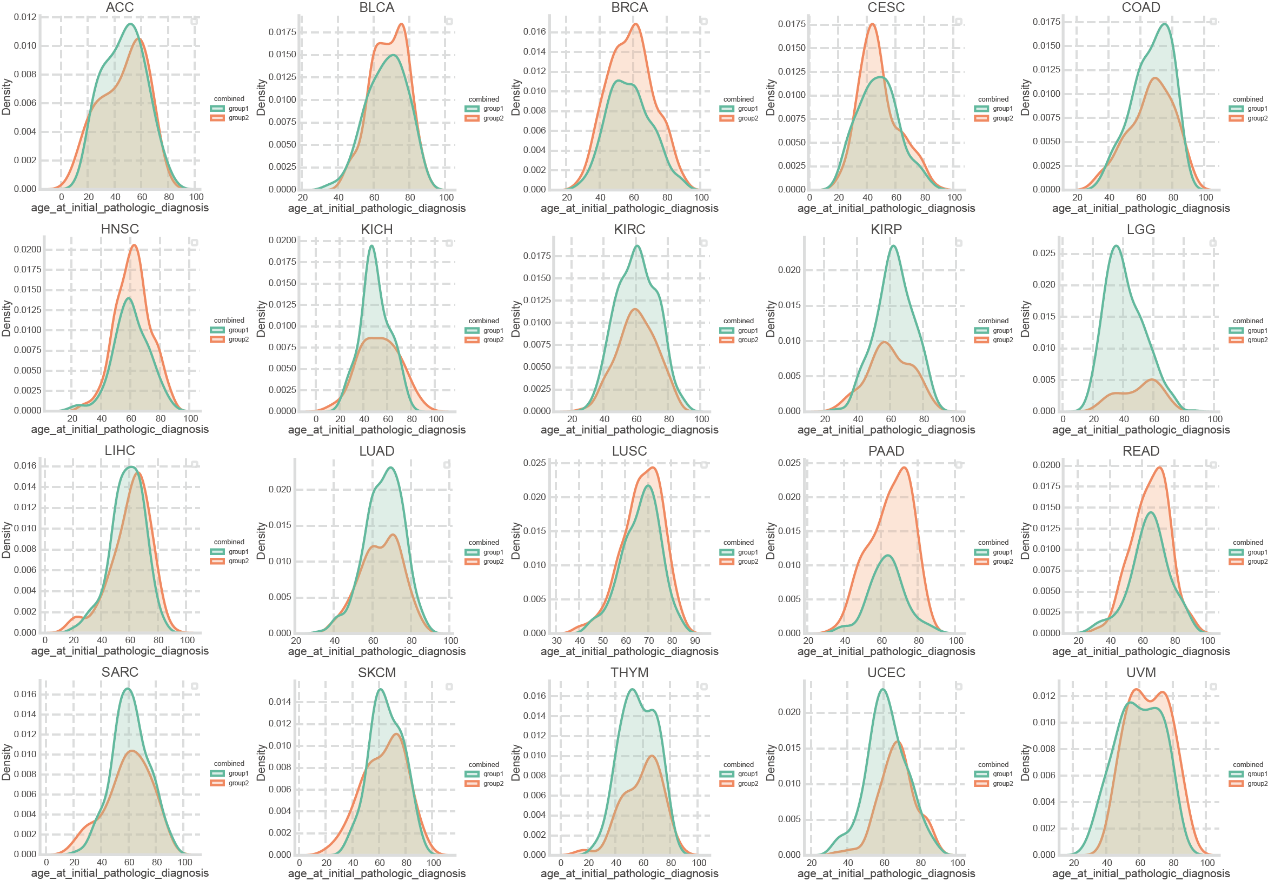
**

**Supplementary Figure 1. Age distribution in ASD-1 and ASD-2 among 20 types of cancer.**

**
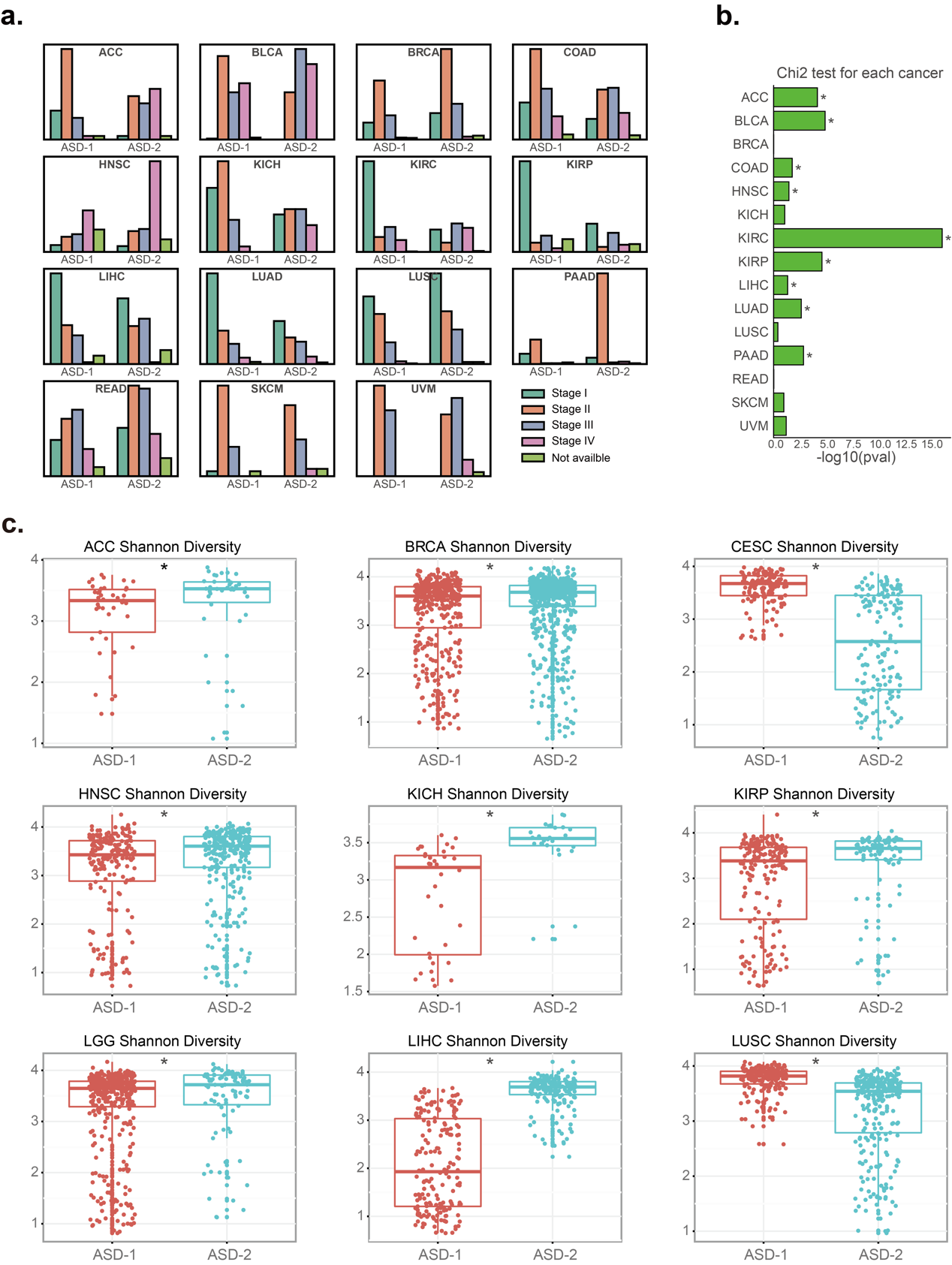
**

**Supplementary Figure 2. Clinical stage distribution and alpha diversity in ASD-1 and ASD-2. a.** Count plot showing the distribution of cancer stages among the subtypes. Five types of cancer (CESC, LGG, SARC, THYM, UCEC) do not have stage information.**b.**Bar plot showing the -log10 p value of a chi-squared test for the distribution of tumor stages among the two subtypes of the 16 types of cancer with stage information. A star symbol indicates a p-value less than 0.05. c. box plots of the significant differences in alpha diversity of tumor microbiomes between the two subtypes, with the test used being the Mann-Whitney test, and the star symbol indicating a p-value less than 0.05.

**
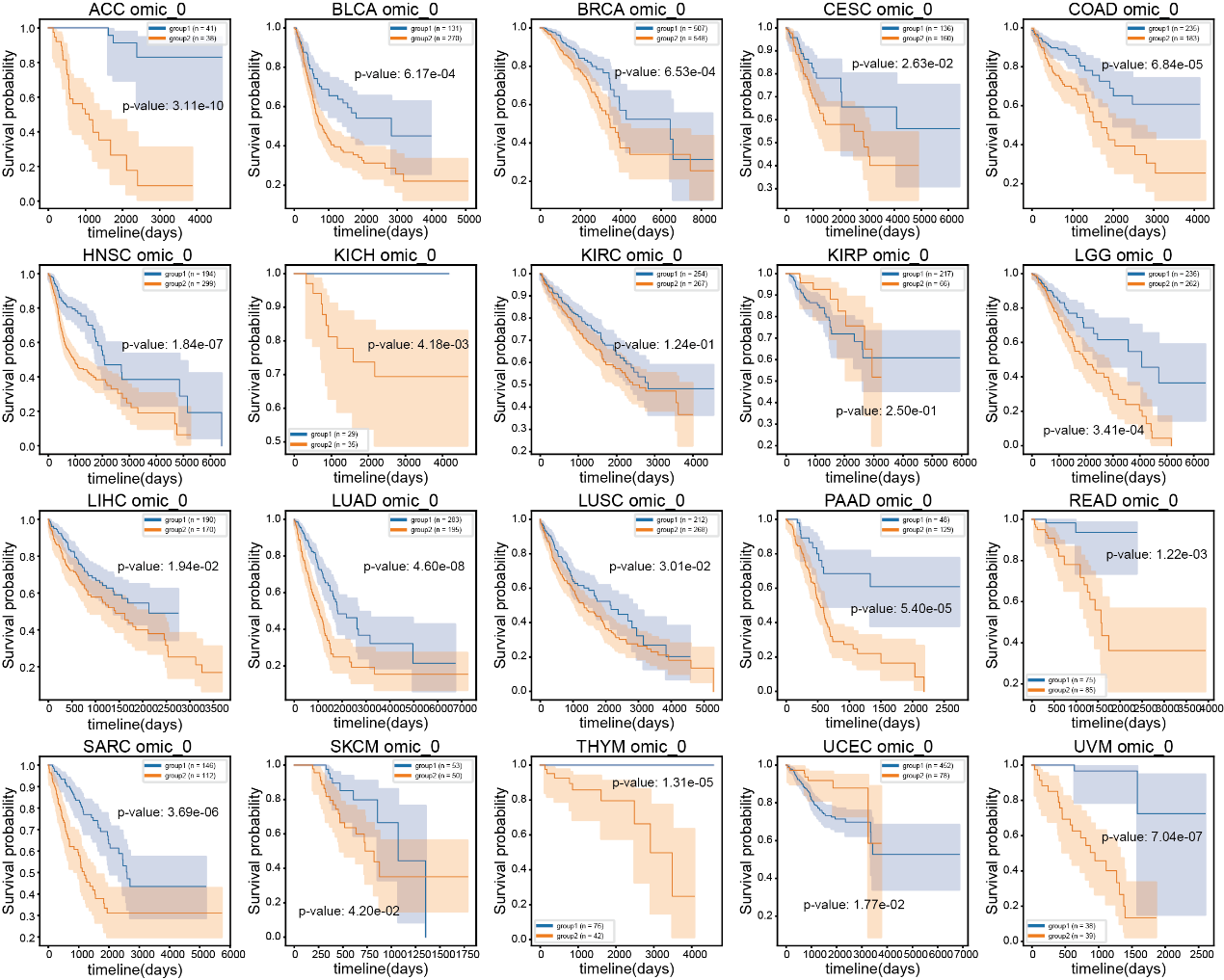
**

**Supplementary Figure 3. Subtyping result only using tumor microbiome.** Kaplan-Meier plots for each type of cancer. The p-value is the result of a log-rank test, which is a statistical test used to compare the survival curves of different groups.

**
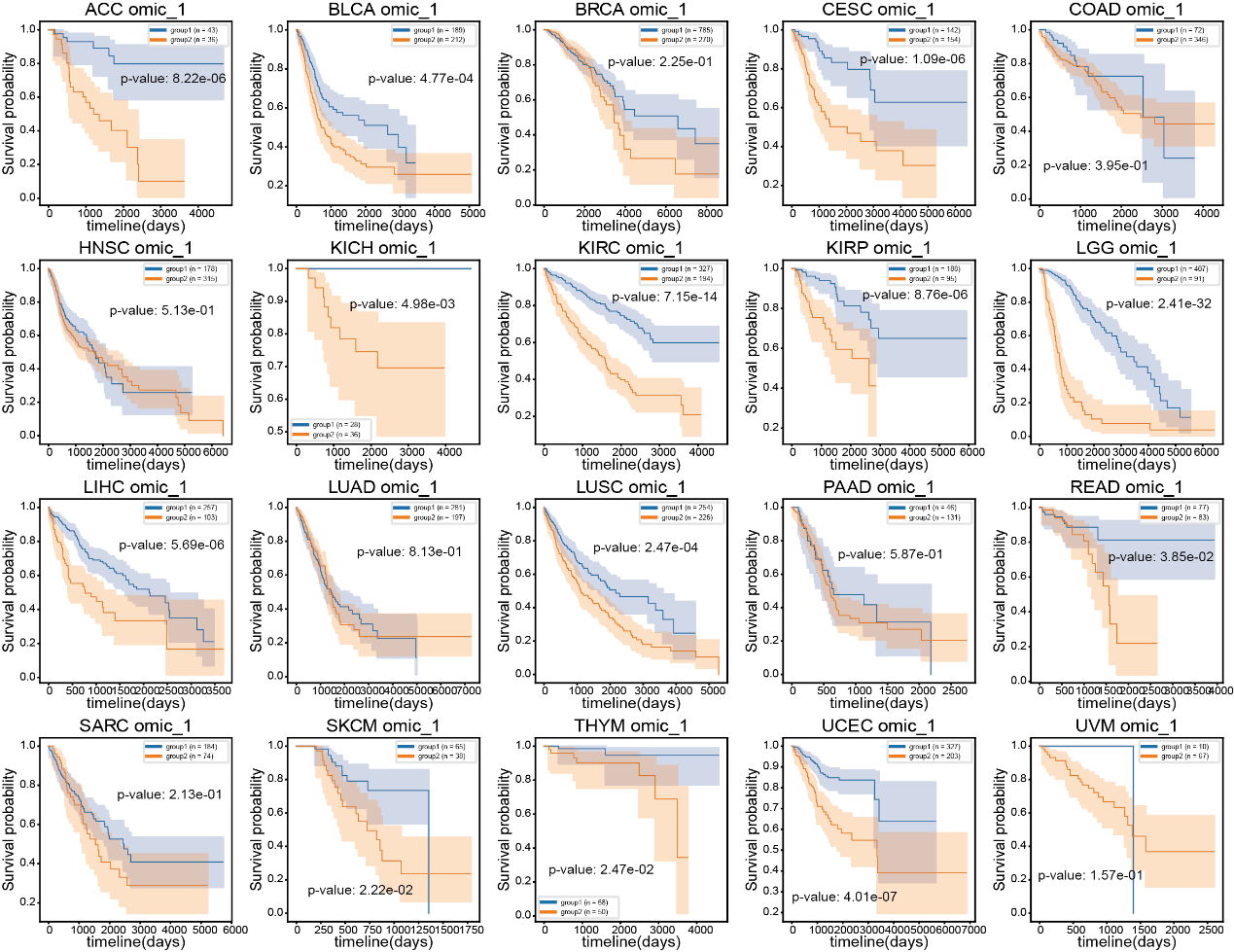
**

**Supplementary Figure 4. Subtyping result only using transcriptome.** Kaplan-Meier plots for each type of cancer. The p-value is the result of a log-rank test, which is a statistical test used to compare the survival curves of different groups.

**
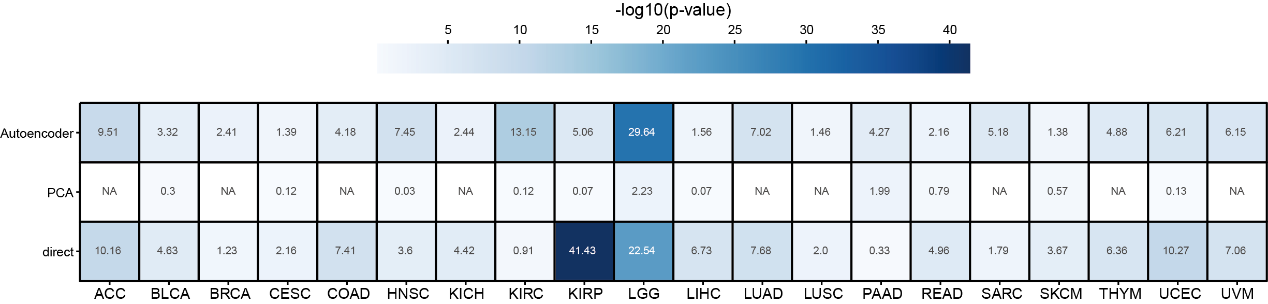
**

**Supplementary Figure 5.** **Heatmap of results obtained using three methods: AE, PCA, and direct feature selection on sample features.** The values in the heatmap represent the -log10 of the p-value from a log-rank test. "NA" indicates that no survival-related features were extracted from at least one of the two omics data sets (RNA-seq and tumor microbiome) for a type of cancer. Direct: Feature selected directly from Cox-PH analysis.

**
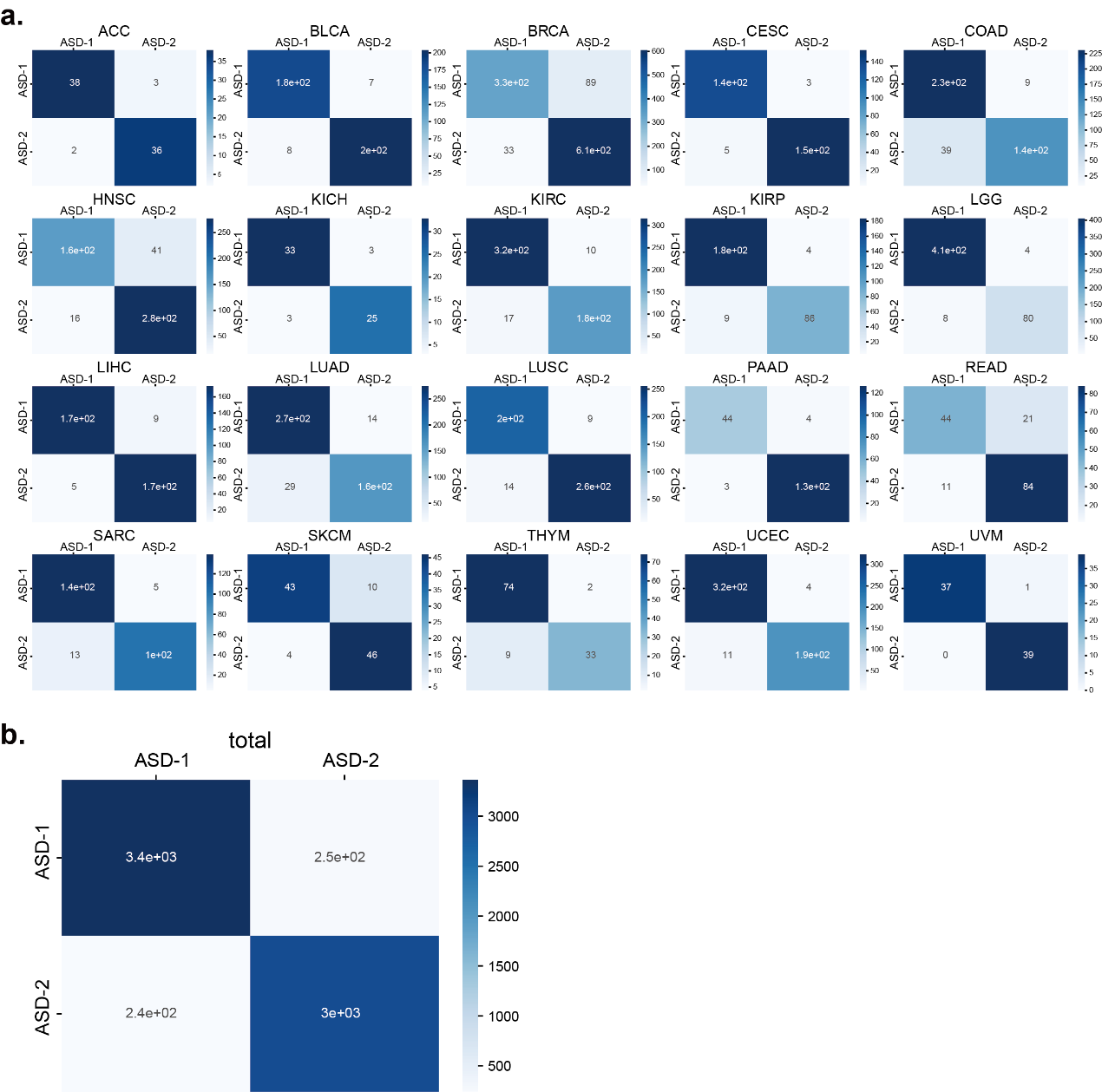
**

**Supplementary Figure 6. Confusion matrices after Random Forest training. a.** Confusion matrices obtained after training Random Forest models for each cancer type individually. **b.** The overall matrix obtained by summing the corresponding positions of all confusion matrices.

**
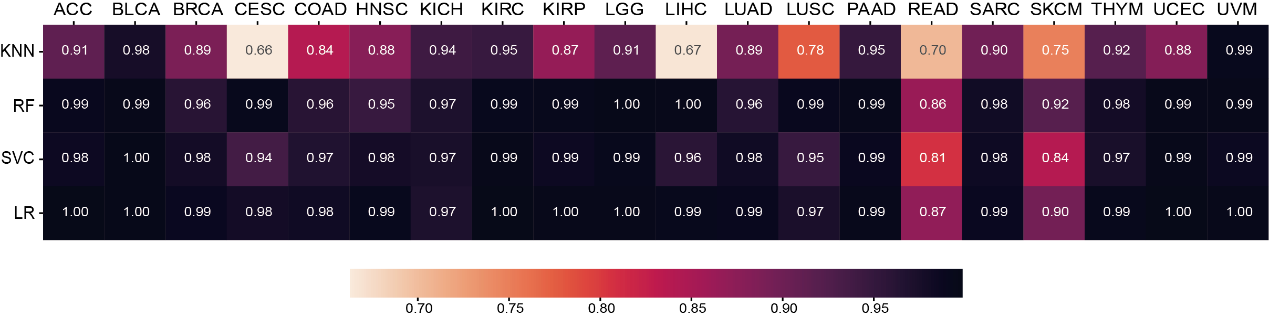
**

**Supplementary Figure 7. The prediction results of subtype.** the area under receiver operating characteristic (AUROC) heatmap of the prediction results of survival subtypes using a leave-one-out method in K Nearest Neighbors (KNN), Random Forest (RF), Support Vector Machine (SVC) and Logistic Regression (LR).

**
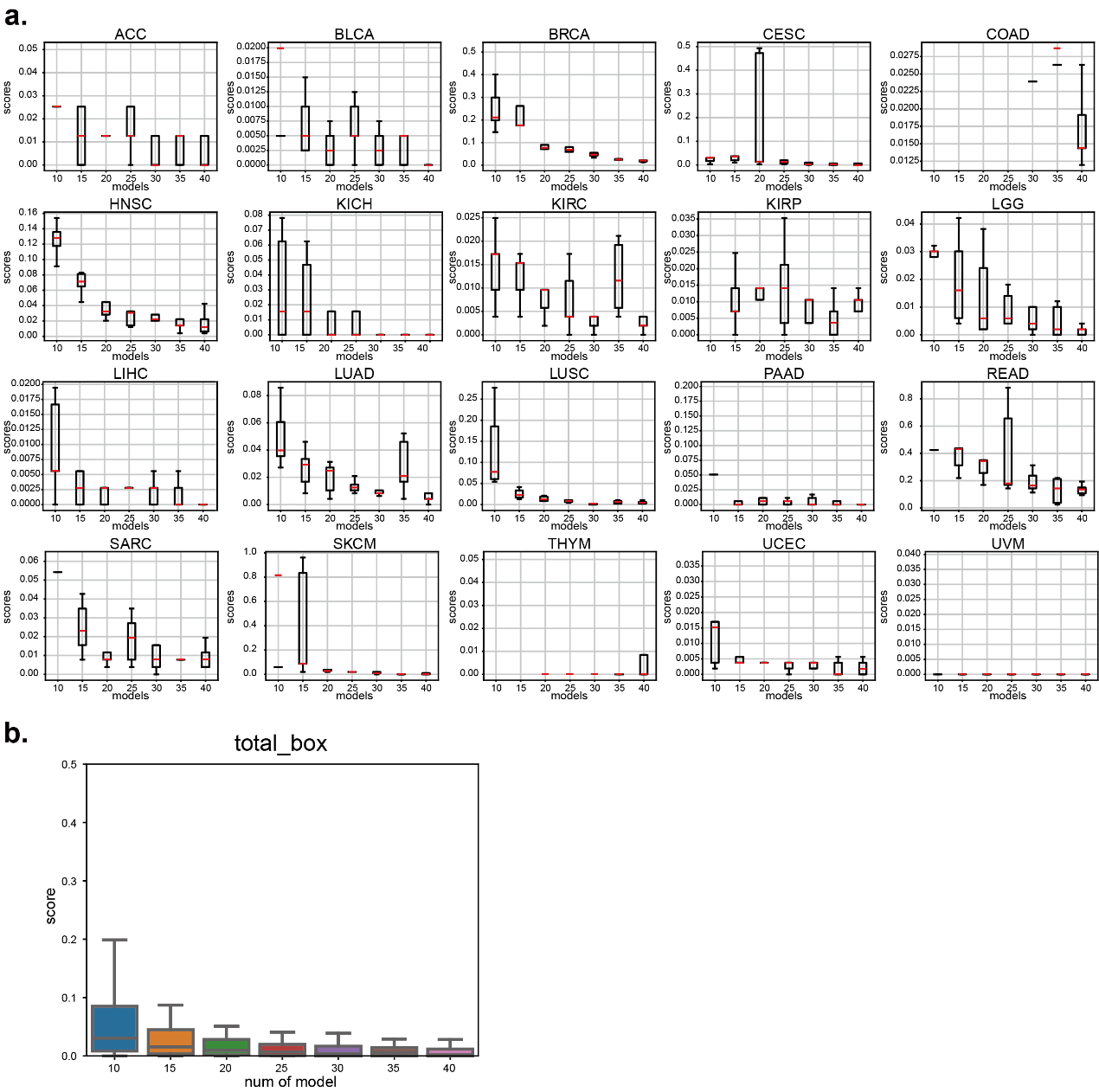
**

**Supplementary Figure 8. Stability of clustering results with different Autoencoder quantities. a.** Boxplot of score distribution for each cancer type. The score is calculated by using the previous result as a reference, determining the number of samples with changed classifications in the current result, and dividing by the total number of samples. Each cancer type is trained with five sets, each containing 40 AEs. The first 5, 10, 15, 20, 25, 30, 35, and 40 AEs are selected for dimensionality reduction, and the score for each set is calculated after filtering and clustering the reduced data. **b.** Boxplot of the overall score distribution. The results for the same AE set across different cancer types are combined to calculate the score, resulting in the final score distribution.

**
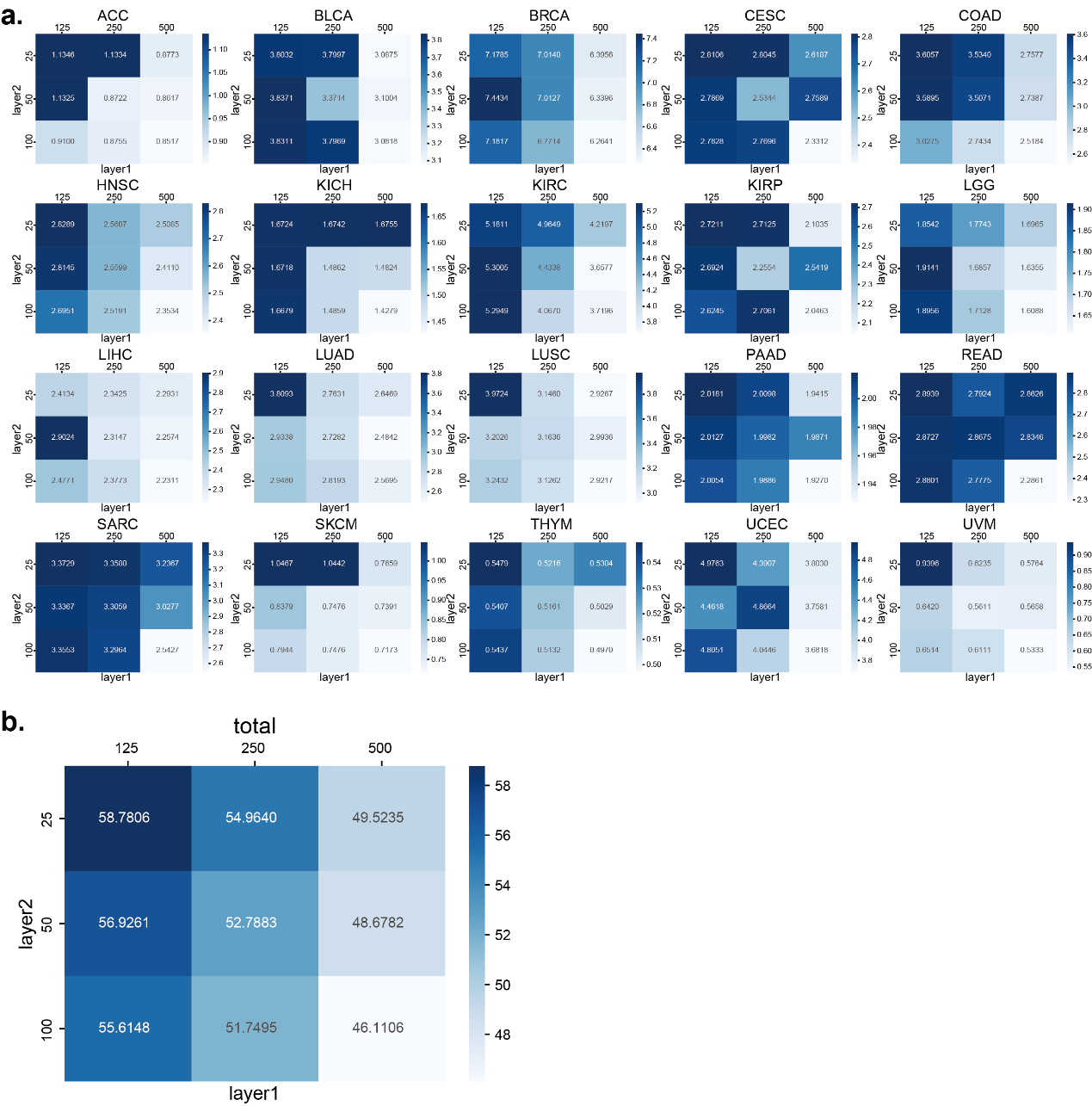
**

**Supplementary Figure 9.** **Mean Squared Error (MSE) matrix on the validation set after Autoencoder training. a.** Autoencoder training results for different cancer types. The matrix shows the MSE on the validation set for each cancer type's autoencoder, where the first layer (layer1) and second layer (layer2) use different neuron counts. The values in each matrix represent the MSE results for specific neuron configurations. **b.** Summary matrix of MSE results for all cancers. The MSE matrices for all cancer types are summed at corresponding positions to obtain the overall MSE distribution.

| **Cancer** | **Stage I** | **Stage II** | **Stage III** | **Stage IV** | **Not available** | **Total** |
| --- | --- | --- | --- | --- | --- | --- |
| ACC | 9 | 37 | 16 | 15 | 2 | 79 |
| BLCA | 1 | 130 | 137 | 131 | 2 | 401 |
| BRCA | 174 | 598 | 240 | 20 | 23 | 1055 |
| CESC | 0 | 0 | 0 | 0 | 296 | 296 |
| COAD | 67 | 163 | 119 | 58 | 11 | 418 |
| HNSC | 24 | 69 | 78 | 255 | 67 | 493 |
| KICH | 19 | 25 | 14 | 6 | 0 | 64 |
| KIRC | 259 | 55 | 122 | 82 | 3 | 521 |
| KIRP | 169 | 20 | 50 | 15 | 29 | 283 |
| LGG | 0 | 0 | 0 | 0 | 498 | 498 |
| LUAD | 252 | 113 | 80 | 26 | 7 | 478 |
| LIHC | 168 | 84 | 80 | 6 | 22 | 360 |
| LUSC | 234 | 152 | 83 | 7 | 4 | 480 |
| PAAD | 21 | 146 | 3 | 4 | 3 | 177 |
| READ | 28 | 49 | 51 | 23 | 9 | 160 |
| SARC | 0 | 0 | 0 | 0 | 258 | 258 |
| SKCM | 2 | 66 | 27 | 3 | 5 | 103 |
| THYM | 0 | 0 | 0 | 0 | 118 | 118 |
| UCEC | 0 | 0 | 0 | 0 | 530 | 530 |
| UVM | 0 | 37 | 35 | 4 | 1 | 77 |

**Supplementary Table 1. Sample counts and stage distribution of each cancer.** ACC, Adrenocortical Carcinoma; BLCA, Bladder Urothelial Carcinoma; BRCA, Breast Invasive Carcinoma; CESC, Cervical Squamous Cell Carcinoma and Endocervical Adenocarcinoma; COAD, Colon Adenocarcinoma; HNSC, Head and Neck Squamous Cell Carcinoma; KICH, Kidney Chromophobe; KIRC, Kidney Renal Clear Cell Carcinoma; KIRP, Kidney Renal Papillary Cell Carcinoma; LGG, Brain Lower Grade Glioma; LIHC, Liver Hepatocellular Carcinoma; LUAD, Lung Adenocarcinoma; LUSC, Lung Squamous Cell Carcinoma; PAAD, Pancreatic Adenocarcinoma; READ, Rectum Adenocarcinoma; SARC, Sarcoma; SKCM, Skin Cutaneous Melanoma; THYM, Thymoma ; UCEC, Uterine Corpus Endometrial Carcinoma; UVM, Uveal Melanoma

**Supplementary Table 2. Top 20 outstanding microbial species with highest contribution of each cancer in subtype prediction.**

| **Adrenocortical Carcinoma** | | |
| --- | --- | --- |
| **Ranking** | **Microbial Types** | **Contribution (×10^­-3^)** |
| 1 | k__Bacteria.p__Firmicutes.c__Bacilli.o__Lactobacillales.f__Streptococcaceae.g__Streptococcus | 22.45 |
| 2 | k__Bacteria.p__Actinobacteria.c__Actinobacteria.o__Corynebacteriales.f__Mycobacteriaceae.g__Mycobacterium | 15.01 |
| 3 | k__Bacteria.p__Tenericutes.c__Mollicutes.o__Acholeplasmatales.f__Acholeplasmataceae.g__Candidatus_Phytoplasma | 11.49 |
| 4 | k__Bacteria.p__Proteobacteria.c__Alphaproteobacteria.o__Rickettsiales.f__Holosporaceae.g__Holospora | 11.19 |
| 5 | k__Bacteria.p__Actinobacteria.c__Actinobacteria.o__Micrococcales.f__Brevibacteriaceae.g__Brevibacterium | 9.58 |
| 6 | k__Bacteria.p__Firmicutes.c__Bacilli.o__Lactobacillales.f__Lactobacillaceae.g__Lactobacillus | 8.11 |
| 7 | k__Bacteria.p__Actinobacteria.c__Actinobacteria.o__Corynebacteriales.f__Corynebacteriaceae.g__Corynebacterium | 7.81 |
| 8 | k__Bacteria.p__Proteobacteria.c__Alphaproteobacteria.o__Rhizobiales.f__Methylocystaceae.g__Methylocystis | 7.44 |
| 9 | k__Bacteria.p__Bacteroidetes.c__Flavobacteriia.o__Flavobacteriales.f__Flavobacteriaceae.g__Lacinutrix | 7.26 |
| 10 | k__Bacteria.p__Tenericutes.c__Mollicutes.o__Entomoplasmatales.f__Entomoplasmataceae.g__Mesoplasma | 7.06 |
| 11 | k__Bacteria.p__Firmicutes.c__Bacilli.o__Lactobacillales.f__Aerococcaceae.g__Aerococcus | 6.48 |
| 12 | k__Bacteria.p__Firmicutes.c__Bacilli.o__Bacillales.f__Bacillaceae.g__Domibacillus | 6.33 |
| 13 | k__Bacteria.p__Proteobacteria.c__Alphaproteobacteria.o__Sphingomonadales.f__Erythrobacteraceae.g__Erythrobacter | 6.19 |
| 14 | k__Bacteria.p__Proteobacteria.c__Gammaproteobacteria.o__Oceanospirillales.f__Oceanospirillaceae.g__Marinomonas | 5.99 |
| 15 | k__Archaea.p__Euryarchaeota.c__Halobacteria.o__Halobacteriales.f__Halobacteriaceae.g__Candidatus_Halobonum | 5.66 |
| 16 | k__Bacteria.p__Proteobacteria.c__Gammaproteobacteria.o__Enterobacteriales.f__Enterobacteriaceae.g__Hafnia | 5.56 |
| 17 | k__Archaea.p__Euryarchaeota.c__Methanobacteria.o__Methanobacteriales.f__Methanobacteriaceae.g__Methanobrevibacter | 5.39 |
| 18 | k__Bacteria.p__Proteobacteria.c__Epsilonproteobacteria.o__Campylobacterales.f__Campylobacteraceae.g__Campylobacter | 5.39 |
| 19 | k__Bacteria.p__Proteobacteria.c__Betaproteobacteria.o__Burkholderiales.g__Ideonella | 5.38 |
| 20 | k__Viruses.f__Polyomaviridae.g__Polyomavirus | 5.29 |

| **Bladder Urothelial Carcinoma** | | |
| --- | --- | --- |
| **Ranking** | **Microbial Types** | **Contribution (×10^­-3^)** |
| 1 | k__Bacteria.p__Proteobacteria.c__Alphaproteobacteria.o__Rhizobiales.f__Hyphomicrobiaceae.g__Prosthecomicrobium | 12.04 |
| 2 | k__Bacteria.p__Firmicutes.c__Clostridia.o__Clostridiales.f__Lachnospiraceae.g__Lachnoclostridium | 8.43 |
| 3 | k__Bacteria.p__Firmicutes.c__Tissierellia.o__Tissierellales.f__Peptoniphilaceae.g__Parvimonas | 8.21 |
| 4 | k__Bacteria.p__Bacteroidetes.c__Cytophagia.o__Cytophagales.f__Flammeovirgaceae.g__Flammeovirga | 7.99 |
| 5 | k__Bacteria.p__Proteobacteria.c__Betaproteobacteria.o__Burkholderiales.f__Sutterellaceae.g__Sutterella | 7.70 |
| 6 | k__Bacteria.p__Proteobacteria.c__Alphaproteobacteria.o__Rhodobacterales.f__Rhodobacteraceae.g__Ruegeria | 7.60 |
| 7 | k__Bacteria.p__Firmicutes.c__Bacilli.o__Lactobacillales.f__Lactobacillaceae.g__Lactobacillus | 6.67 |
| 8 | k__Viruses.f__Nimaviridae.g__Whispovirus | 6.08 |
| 9 | k__Bacteria.p__Spirochaetes.c__Spirochaetia.o__Brachyspirales.f__Brachyspiraceae.g__Brachyspira | 6.08 |
| 10 | k__Bacteria.p__Proteobacteria.c__Gammaproteobacteria.o__Oceanospirillales.f__Alcanivoracaceae.g__Alcanivorax | 5.93 |
| 11 | k__Archaea.p__Thaumarchaeota.g__Candidatus_Nitrosopelagicus | 5.68 |
| 12 | k__Bacteria.p__Firmicutes.c__Clostridia.o__Clostridiales.f__Ruminococcaceae.g__Mageeibacillus | 5.33 |
| 13 | k__Bacteria.p__Bacteroidetes.c__Bacteroidia.o__Bacteroidales.f__Porphyromonadaceae.g__Dysgonomonas | 5.22 |
| 14 | k__Bacteria.p__Bacteroidetes.c__Cytophagia.o__Cytophagales.f__Cytophagaceae.g__Anditalea | 5.09 |
| 15 | k__Bacteria.p__Proteobacteria.c__Alphaproteobacteria.o__Rhizobiales.f__Bradyrhizobiaceae.g__Rhodopseudomonas | 5.06 |
| 16 | k__Bacteria.p__Proteobacteria.c__Gammaproteobacteria.o__Pasteurellales.f__Pasteurellaceae.g__Mannheimia | 4.97 |
| 17 | k__Bacteria.p__Bacteroidetes.c__Cytophagia.o__Cytophagales.f__Cyclobacteriaceae.g__Indibacter | 4.94 |
| 18 | k__Bacteria.p__Proteobacteria.c__Gammaproteobacteria.o__Pasteurellales.f__Pasteurellaceae.g__Gallibacterium | 4.70 |
| 19 | k__Bacteria.p__Proteobacteria.c__Deltaproteobacteria.o__Desulfuromonadales.f__Geobacteraceae.g__Geobacter | 4.66 |
| 20 | k__Viruses.o__Herpesvirales.f__Herpesviridae.g__Roseolovirus | 4.47 |

| **Breast Invasive Carcinoma** | | |
| --- | --- | --- |
| **Ranking** | **Microbial Types** | **Contribution (×10^­-3^)** |
| 1 | k__Bacteria.p__Proteobacteria.c__Gammaproteobacteria.o__Aeromonadales.f__Succinivibrionaceae.g__Succinimonas | 20.50 |
| 2 | k__Bacteria.p__Proteobacteria.c__Betaproteobacteria.o__Neisseriales.f__Chromobacteriaceae.g__Pseudogulbenkiania | 14.72 |
| 3 | k__Bacteria.p__Firmicutes.c__Clostridia.o__Clostridiales.f__Peptostreptococcaceae.g__Clostridioides | 12.83 |
| 4 | k__Bacteria.p__Actinobacteria.c__Actinobacteria.o__Bifidobacteriales.f__Bifidobacteriaceae.g__Scardovia | 11.03 |
| 5 | k__Bacteria.p__Proteobacteria.c__Betaproteobacteria.o__Burkholderiales.f__Comamonadaceae.g__Curvibacter | 10.41 |
| 6 | k__Bacteria.p__Proteobacteria.c__Betaproteobacteria.o__Burkholderiales.f__Comamonadaceae.g__Simplicispira | 10.13 |
| 7 | k__Bacteria.p__Proteobacteria.c__Alphaproteobacteria.o__Pelagibacterales.f__Pelagibacteraceae.g__Candidatus_Pelagibacter | 8.29 |
| 8 | k__Bacteria.p__Proteobacteria.c__Gammaproteobacteria.o__Enterobacteriales.f__Enterobacteriaceae.g__Edwardsiella | 7.87 |
| 9 | k__Bacteria.p__Bacteroidetes.c__Cytophagia.o__Cytophagales.f__Cyclobacteriaceae.g__Indibacter | 7.00 |
| 10 | k__Bacteria.p__Actinobacteria.c__Actinobacteria.o__Micromonosporales.f__Micromonosporaceae.g__Catenuloplanes | 6.24 |
| 11 | k__Viruses.o__Herpesvirales.f__Herpesviridae.g__Simplexvirus | 6.01 |
| 12 | k__Bacteria.p__Actinobacteria.c__Actinobacteria.o__Micromonosporales.f__Micromonosporaceae.g__Salinispora | 5.89 |
| 13 | k__Bacteria.p__Actinobacteria.c__Actinobacteria.o__Micrococcales.f__Demequinaceae.g__Lysinimicrobium | 5.86 |
| 14 | k__Bacteria.p__Proteobacteria.c__Gammaproteobacteria.o__Enterobacteriales.f__Enterobacteriaceae.g__Xenorhabdus | 5.84 |
| 15 | k__Bacteria.p__Bacteroidetes.c__Bacteroidia.o__Bacteroidales.f__Porphyromonadaceae.g__Tannerella | 5.82 |
| 16 | k__Bacteria.p__Actinobacteria.c__Actinobacteria.o__Micrococcales.f__Microbacteriaceae.g__Leucobacter | 5.52 |
| 17 | k__Bacteria.p__Proteobacteria.c__Deltaproteobacteria.o__Desulfobacterales.f__Desulfobulbaceae.g__Desulfotalea | 5.33 |
| 18 | k__Bacteria.p__Bacteroidetes.c__Cytophagia.o__Cytophagales.f__Flammeovirgaceae.g__Roseivirga | 4.74 |
| 19 | k__Bacteria.p__Bacteroidetes.c__Chitinophagia.o__Chitinophagales.f__Chitinophagaceae.g__Sediminibacterium | 4.69 |
| 20 | k__Bacteria.p__Proteobacteria.c__Gammaproteobacteria.o__Enterobacteriales.f__Enterobacteriaceae.g__Shigella | 4.58 |

| **Cervical Squamous Cell Carcinoma and Endocervical Adenocarcinoma** | | |
| --- | --- | --- |
| **Ranking** | **Microbial Types** | **Contribution (×10^­-3^)** |
| 1 | k__Bacteria.p__Proteobacteria.c__Alphaproteobacteria.o__Rhizobiales.f__Bartonellaceae.g__Bartonella | 29.05 |
| 2 | k__Archaea.p__Euryarchaeota.c__Methanomicrobia.o__Methanosarcinales.f__Methanosarcinaceae.g__Methanosarcina | 27.84 |
| 3 | k__Bacteria.p__Planctomycetes.c__Planctomycetia.o__Planctomycetales.f__Planctomycetaceae.g__Gemmata | 23.95 |
| 4 | k__Bacteria.p__Firmicutes.c__Clostridia.o__Thermoanaerobacterales.f__Thermoanaerobacteraceae.g__Thermoanaerobacter | 22.39 |
| 5 | k__Bacteria.p__Proteobacteria.c__Alphaproteobacteria.o__Rickettsiales.f__Rickettsiaceae.g__Rickettsia | 21.45 |
| 6 | k__Bacteria.p__Tenericutes.c__Mollicutes.o__Mycoplasmatales.f__Mycoplasmataceae.g__Mycoplasma | 21.16 |
| 7 | k__Bacteria.p__Firmicutes.c__Clostridia.o__Clostridiales.g__Epulopiscium | 20.83 |
| 8 | k__Bacteria.p__Proteobacteria.c__Gammaproteobacteria.o__Vibrionales.f__Vibrionaceae.g__Grimontia | 20.51 |
| 9 | k__Bacteria.p__Proteobacteria.c__Betaproteobacteria.o__Burkholderiales.f__Oxalobacteraceae.g__Herbaspirillum | 19.32 |
| 10 | k__Bacteria.p__Proteobacteria.c__Gammaproteobacteria.o__Xanthomonadales.f__Rhodanobacteraceae.g__Luteibacter | 19.20 |
| 11 | k__Bacteria.p__Proteobacteria.c__Epsilonproteobacteria.o__Campylobacterales.f__Helicobacteraceae.g__Helicobacter | 18.19 |
| 12 | k__Bacteria.p__Proteobacteria.c__Betaproteobacteria.o__Burkholderiales.f__Alcaligenaceae.g__Brackiella | 17.61 |
| 13 | k__Bacteria.p__Proteobacteria.c__Gammaproteobacteria.o__Alteromonadales.f__Pseudoalteromonadaceae.g__Pseudoalteromonas | 17.40 |
| 14 | k__Bacteria.p__Proteobacteria.c__Gammaproteobacteria.o__Enterobacteriales.f__Enterobacteriaceae.g__Buchnera | 16.82 |
| 15 | k__Bacteria.p__Proteobacteria.c__Gammaproteobacteria.o__Vibrionales.f__Vibrionaceae.g__Vibrio | 16.28 |
| 16 | k__Bacteria.p__Firmicutes.c__Bacilli.o__Bacillales.f__Listeriaceae.g__Listeria | 14.93 |
| 17 | k__Bacteria.p__Proteobacteria.c__Gammaproteobacteria.o__Pasteurellales.f__Pasteurellaceae.g__Histophilus | 13.11 |
| 18 | k__Viruses.f__Polydnaviridae.g__Ichnovirus | 12.83 |
| 19 | k__Bacteria.p__Spirochaetes.c__Spirochaetia.o__Spirochaetales.f__Borreliaceae.g__Borrelia | 12.72 |
| 20 | k__Bacteria.p__Cyanobacteria.o__Prochlorales.f__Prochlorococcaceae.g__Prochlorococcus | 12.71 |

| **Colon Adenocarcinoma** | | |
| --- | --- | --- |
| **Ranking** | **Microbial Types** | **Contribution (×10^­-3^)** |
| 1 | k__Bacteria.p__Proteobacteria.c__Gammaproteobacteria.o__Alteromonadales.f__Pseudoalteromonadaceae.g__Algicola | 9.28 |
| 2 | k__Bacteria.p__Cyanobacteria.o__Prochlorales.f__Prochlorococcaceae.g__Prochlorococcus | 7.52 |
| 3 | k__Bacteria.p__Proteobacteria.c__Gammaproteobacteria.o__Aeromonadales.f__Succinivibrionaceae.g__Succinimonas | 6.43 |
| 4 | k__Bacteria.p__Bacteroidetes.c__Cytophagia.o__Cytophagales.f__Cyclobacteriaceae.g__Indibacter | 6.19 |
| 5 | k__Bacteria.p__Firmicutes.c__Clostridia.o__Thermoanaerobacterales.f__Thermoanaerobacteraceae.g__Caldanaerobacter | 5.93 |
| 6 | k__Bacteria.p__Actinobacteria.c__Actinobacteria.o__Micrococcales.f__Dermabacteraceae.g__Brachybacterium | 5.92 |
| 7 | k__Bacteria.p__Actinobacteria.c__Actinobacteria.o__Corynebacteriales.f__Nocardiaceae.g__Nocardia | 5.30 |
| 8 | k__Bacteria.p__Proteobacteria.c__Gammaproteobacteria.o__Vibrionales.f__Vibrionaceae.g__Vibrio | 5.11 |
| 9 | k__Bacteria.p__Actinobacteria.c__Actinobacteria.o__Micrococcales.f__Micrococcaceae.g__Paenarthrobacter | 4.94 |
| 10 | k__Bacteria.p__Bacteroidetes.c__Bacteroidia.o__Bacteroidales.f__Prolixibacteraceae.g__Sunxiuqinia | 4.78 |
| 11 | k__Bacteria.p__Actinobacteria.c__Actinobacteria.o__Micrococcales.f__Intrasporangiaceae.g__Terrabacter | 4.74 |
| 12 | k__Bacteria.p__Proteobacteria.c__Gammaproteobacteria.o__Aeromonadales.f__Aeromonadaceae.g__Oceanimonas | 4.46 |
| 13 | k__Bacteria.p__Tenericutes.c__Mollicutes.o__Mycoplasmatales.f__Mycoplasmataceae.g__Mycoplasma | 4.18 |
| 14 | k__Bacteria.p__Proteobacteria.c__Alphaproteobacteria.o__Rhizobiales.f__Methylobacteriaceae.g__Microvirga | 3.81 |
| 15 | k__Bacteria.p__Proteobacteria.c__Alphaproteobacteria.o__Rhizobiales.f__Rhizobiaceae.g__Sinorhizobium | 3.77 |
| 16 | k__Bacteria.p__Cyanobacteria.o__Chroococcales.g__Chamaesiphon | 3.69 |
| 17 | k__Bacteria.p__Firmicutes.c__Bacilli.o__Bacillales.g__Acidibacillus | 3.67 |
| 18 | k__Bacteria.p__Proteobacteria.c__Deltaproteobacteria.o__Desulfobacterales.f__Desulfobulbaceae.g__Desulfotalea | 3.57 |
| 19 | k__Bacteria.p__Firmicutes.c__Bacilli.o__Bacillales.f__Paenibacillaceae.g__Gorillibacterium | 3.53 |
| 20 | k__Bacteria.p__Actinobacteria.c__Actinobacteria.o__Micrococcales.f__Micrococcaceae.g__Kocuria | 3.42 |

| **Head and Neck Squamous Cell Carcinoma** | | |
| --- | --- | --- |
| **Ranking** | **Microbial Types** | **Contribution (×10^­-3^)** |
| 1 | k__Viruses.f__Papillomaviridae.g__Alphapapillomavirus | 15.70 |
| 2 | k__Bacteria.p__Firmicutes.c__Clostridia.o__Clostridiales.f__Clostridiaceae.g__Clostridium | 10.53 |
| 3 | k__Bacteria.p__Firmicutes.c__Tissierellia.o__Tissierellales.f__Peptoniphilaceae.g__Parvimonas | 10.37 |
| 4 | k__Bacteria.p__Proteobacteria.c__Gammaproteobacteria.o__Pasteurellales.f__Pasteurellaceae.g__Necropsobacter | 9.19 |
| 5 | k__Bacteria.p__Bacteroidetes.c__Flavobacteriia.o__Flavobacteriales.f__Flavobacteriaceae.g__Flavobacterium | 7.76 |
| 6 | k__Bacteria.p__Proteobacteria.c__Gammaproteobacteria.o__Aeromonadales.f__Succinivibrionaceae.g__Succinimonas | 6.50 |
| 7 | k__Bacteria.p__Bacteroidetes.c__Flavobacteriia.o__Flavobacteriales.f__Flavobacteriaceae.g__Ornithobacterium | 6.35 |
| 8 | k__Bacteria.p__Proteobacteria.c__Gammaproteobacteria.o__Alteromonadales.f__Colwelliaceae.g__Colwellia | 5.82 |
| 9 | k__Bacteria.p__Proteobacteria.c__Gammaproteobacteria.o__Pasteurellales.f__Pasteurellaceae.g__Gallibacterium | 5.56 |
| 10 | k__Bacteria.p__Tenericutes.c__Mollicutes.o__Mycoplasmatales.f__Mycoplasmataceae.g__Ureaplasma | 5.54 |
| 11 | k__Bacteria.p__Actinobacteria.c__Actinobacteria.o__Corynebacteriales.f__Dietziaceae.g__Dietzia | 4.37 |
| 12 | k__Bacteria.p__Bacteroidetes.c__Cytophagia.o__Cytophagales.f__Hymenobacteraceae.g__Hymenobacter | 4.35 |
| 13 | k__Bacteria.p__Firmicutes.c__Clostridia.o__Clostridiales.f__Lachnospiraceae.g__Lachnoclostridium | 4.18 |
| 14 | k__Viruses.o__Herpesvirales.f__Herpesviridae.g__Mardivirus | 4.10 |
| 15 | k__Bacteria.p__Proteobacteria.c__Alphaproteobacteria.o__Rhodobacterales.f__Rhodobacteraceae.g__Ruegeria | 3.93 |
| 16 | k__Bacteria.p__Proteobacteria.c__Betaproteobacteria.o__Burkholderiales.f__Oxalobacteraceae.g__Collimonas | 3.92 |
| 17 | k__Bacteria.p__Proteobacteria.c__Alphaproteobacteria.o__Caulobacterales.f__Caulobacteraceae.g__Phenylobacterium | 3.84 |
| 18 | k__Bacteria.p__Proteobacteria.c__Alphaproteobacteria.o__Rickettsiales.f__Rickettsiaceae.g__Rickettsia | 3.77 |
| 19 | k__Bacteria.p__Proteobacteria.c__Alphaproteobacteria.o__Rhodobacterales.f__Rhodobacteraceae.g__Nautella | 3.62 |
| 20 | k__Bacteria.p__Proteobacteria.c__Betaproteobacteria.o__Burkholderiales.f__Oxalobacteraceae.g__Herbaspirillum | 3.51 |

| **Kidney Chromophobe** | | |
| --- | --- | --- |
| **Ranking** | **Microbial Types** | **Contribution (×10^­-3^)** |
| 1 | k__Bacteria.p__Proteobacteria.c__Epsilonproteobacteria.o__Campylobacterales.f__Helicobacteraceae.g__Helicobacter | 28.29 |
| 2 | k__Bacteria.p__Proteobacteria.c__Gammaproteobacteria.o__Enterobacteriales.f__Enterobacteriaceae.g__Escherichia | 24.45 |
| 3 | k__Bacteria.p__Proteobacteria.c__Betaproteobacteria.o__Burkholderiales.f__Alcaligenaceae.g__Brackiella | 23.36 |
| 4 | k__Archaea.p__Crenarchaeota.c__Thermoprotei.o__Sulfolobales.f__Sulfolobaceae.g__Sulfolobus | 20.77 |
| 5 | k__Bacteria.p__Proteobacteria.c__Deltaproteobacteria.o__Desulfovibrionales.f__Desulfovibrionaceae.g__Lawsonia | 20.07 |
| 6 | k__Bacteria.p__Fusobacteria.c__Fusobacteriia.o__Fusobacteriales.f__Leptotrichiaceae.g__Sneathia | 19.99 |
| 7 | k__Bacteria.p__Proteobacteria.c__Gammaproteobacteria.o__Legionellales.f__Legionellaceae.g__Legionella | 18.23 |
| 8 | k__Bacteria.p__Proteobacteria.c__Alphaproteobacteria.o__Rhodospirillales.f__Acetobacteraceae.g__Saccharibacter | 17.43 |
| 9 | k__Bacteria.p__Proteobacteria.c__Gammaproteobacteria.o__Enterobacteriales.f__Enterobacteriaceae.g__Raoultella | 16.71 |
| 10 | k__Bacteria.p__Proteobacteria.c__Alphaproteobacteria.o__Rhodobacterales.f__Rhodobacteraceae.g__Loktanella | 16.22 |
| 11 | k__Viruses.f__Phycodnaviridae.g__Prymnesiovirus | 15.77 |
| 12 | k__Bacteria.p__Firmicutes.c__Bacilli.o__Bacillales.g__Acidibacillus | 15.59 |
| 13 | k__Bacteria.p__Proteobacteria.c__Alphaproteobacteria.o__Caulobacterales.f__Caulobacteraceae.g__Caulobacter | 15.34 |
| 14 | k__Bacteria.p__Proteobacteria.c__Gammaproteobacteria.o__Alteromonadales.f__Pseudoalteromonadaceae.g__Pseudoalteromonas | 14.12 |
| 15 | k__Bacteria.p__Bacteroidetes.c__Flavobacteriia.o__Flavobacteriales.f__Flavobacteriaceae.g__Psychroserpens | 13.96 |
| 16 | k__Bacteria.p__Bacteroidetes.c__Bacteroidia.o__Bacteroidales.f__Prevotellaceae.g__Prevotella | 12.78 |
| 17 | k__Viruses.f__Partitiviridae.g__Betapartitivirus | 12.41 |
| 18 | k__Bacteria.p__Cyanobacteria.o__Chroococcales.g__Cyanothece | 12.18 |
| 19 | k__Bacteria.p__Bacteroidetes.c__Flavobacteriia.o__Flavobacteriales.f__Flavobacteriaceae.g__Elizabethkingia | 11.79 |
| 20 | k__Bacteria.p__Fusobacteria.c__Fusobacteriia.o__Fusobacteriales.f__Leptotrichiaceae.g__Leptotrichia | 11.74 |

| **Kidney Renal Clear Cell Carcinoma** | | |
| --- | --- | --- |
| **Ranking** | **Microbial Types** | **Contribution (×10^­-3^)** |
| 1 | k__Viruses.o__Herpesvirales.f__Herpesviridae.g__Simplexvirus | 14.07 |
| 2 | k__Bacteria.p__Fibrobacteres.c__Chitinivibrionia.o__Chitinivibrionales.f__Chitinivibrionaceae.g__Chitinivibrio | 8.19 |
| 3 | k__Archaea.p__Thaumarchaeota.g__Candidatus_Nitrosopelagicus | 7.75 |
| 4 | k__Bacteria.p__Cyanobacteria.o__Stigonematales.g__Mastigocoleus | 7.47 |
| 5 | k__Bacteria.p__Proteobacteria.c__Alphaproteobacteria.o__Rhodobacterales.f__Hyphomonadaceae.g__Maricaulis | 7.32 |
| 6 | k__Bacteria.p__Bacteroidetes.c__Cytophagia.o__Cytophagales.f__Cytophagaceae.g__Cytophaga | 6.43 |
| 7 | k__Bacteria.p__Firmicutes.c__Bacilli.o__Lactobacillales.f__Streptococcaceae.g__Streptococcus | 6.31 |
| 8 | k__Bacteria.p__Actinobacteria.c__Actinobacteria.o__Corynebacteriales.f__Gordoniaceae.g__Gordonia | 5.88 |
| 9 | k__Bacteria.p__Actinobacteria.c__Actinobacteria.o__Micrococcales.f__Microbacteriaceae.g__Plantibacter | 5.08 |
| 10 | k__Bacteria.p__Proteobacteria.c__Gammaproteobacteria.o__Aeromonadales.f__Aeromonadaceae.g__Aeromonas | 4.96 |
| 11 | k__Bacteria.p__Actinobacteria.c__Actinobacteria.o__Micrococcales.f__Beutenbergiaceae.g__Beutenbergia | 4.89 |
| 12 | k__Bacteria.p__Actinobacteria.c__Actinobacteria.o__Micrococcales.f__Brevibacteriaceae.g__Brevibacterium | 4.72 |
| 13 | k__Bacteria.p__Proteobacteria.c__Deltaproteobacteria.o__Desulfobacterales.f__Desulfobulbaceae.g__Desulfotalea | 4.69 |
| 14 | k__Bacteria.p__Bacteroidetes.c__Cytophagia.o__Cytophagales.f__Cyclobacteriaceae.g__Indibacter | 4.57 |
| 15 | k__Viruses.f__Phycodnaviridae.g__Prymnesiovirus | 4.37 |
| 16 | k__Bacteria.p__Chloroflexi.c__Chloroflexia.o__Chloroflexales.f__Roseiflexaceae.g__Roseiflexus | 4.09 |
| 17 | k__Bacteria.p__Proteobacteria.c__Alphaproteobacteria.o__Sphingomonadales.f__Erythrobacteraceae.g__Altererythrobacter | 3.99 |
| 18 | k__Bacteria.p__Proteobacteria.c__Gammaproteobacteria.o__Vibrionales.f__Vibrionaceae.g__Vibrio | 3.93 |
| 19 | k__Bacteria.p__Firmicutes.c__Erysipelotrichia.o__Erysipelotrichales.f__Erysipelotrichaceae.g__Bulleidia | 3.76 |
| 20 | k__Archaea.p__Crenarchaeota.c__Thermoprotei.o__Sulfolobales.f__Sulfolobaceae.g__Sulfolobus | 3.75 |

| **Kidney Renal Papillary Cell Carcinoma** | | |
| --- | --- | --- |
| **Ranking** | **Microbial Types** | **Contribution (×10^­-3^)** |
| 1 | k__Bacteria.p__Bacteroidetes.c__Flavobacteriia.o__Flavobacteriales.f__Flavobacteriaceae.g__Apibacter | 9.42 |
| 2 | k__Archaea.p__Thaumarchaeota.g__Candidatus_Nitrosopelagicus | 9.05 |
| 3 | k__Viruses.o__Herpesvirales.f__Herpesviridae.g__Simplexvirus | 8.95 |
| 4 | k__Bacteria.p__Bacteroidetes.c__Cytophagia.o__Cytophagales.f__Cyclobacteriaceae.g__Indibacter | 8.36 |
| 5 | k__Bacteria.p__Proteobacteria.c__Gammaproteobacteria.o__Vibrionales.f__Vibrionaceae.g__Vibrio | 7.83 |
| 6 | k__Bacteria.p__Proteobacteria.c__Gammaproteobacteria.o__Enterobacteriales.f__Enterobacteriaceae.g__Edwardsiella | 6.25 |
| 7 | k__Bacteria.p__Spirochaetes.c__Spirochaetia.o__Spirochaetales.f__Spirochaetaceae.g__Treponema | 6.13 |
| 8 | k__Bacteria.p__Proteobacteria.c__Alphaproteobacteria.o__Rhodobacterales.f__Rhodobacteraceae.g__Actibacterium | 5.85 |
| 9 | k__Bacteria.p__Proteobacteria.c__Gammaproteobacteria.o__Oceanospirillales.f__Oceanospirillaceae.g__Oceanobacter | 5.58 |
| 10 | k__Bacteria.p__Proteobacteria.c__Alphaproteobacteria.o__Rhodospirillales.f__Acetobacteraceae.g__Komagataeibacter | 5.30 |
| 11 | k__Bacteria.p__Proteobacteria.c__Alphaproteobacteria.o__Rhodobacterales.f__Rhodobacteraceae.g__Loktanella | 5.16 |
| 12 | k__Bacteria.p__Proteobacteria.c__Betaproteobacteria.o__Burkholderiales.f__Burkholderiaceae.g__Paraburkholderia | 5.07 |
| 13 | k__Bacteria.p__Bacteroidetes.c__Flavobacteriia.o__Flavobacteriales.f__Flavobacteriaceae.g__Chryseobacterium | 4.97 |
| 14 | k__Viruses.f__Bromoviridae.g__Bromovirus | 4.75 |
| 15 | k__Bacteria.p__Bacteroidetes.c__Flavobacteriia.o__Flavobacteriales.f__Flavobacteriaceae.g__Gelidibacter | 4.64 |
| 16 | k__Bacteria.p__Thermotogae.c__Thermotogae.o__Petrotogales.g__Marinitoga | 4.51 |
| 17 | k__Viruses.f__Polyomaviridae.g__Polyomavirus | 4.41 |
| 18 | k__Bacteria.p__Nitrospirae.c__Nitrospira.o__Nitrospirales.f__Nitrospiraceae.g__Nitrospira | 4.40 |
| 19 | k__Bacteria.p__Tenericutes.c__Mollicutes.o__Mycoplasmatales.f__Mycoplasmataceae.g__Ureaplasma | 4.29 |
| 20 | k__Viruses.f__Phycodnaviridae.g__Prymnesiovirus | 4.25 |

| **Brain Lower Grade Glioma** | | |
| --- | --- | --- |
| **Ranking** | **Microbial Types** | **Contribution (×10^­-3^)** |
| 1 | k__Bacteria.p__Firmicutes.c__Clostridia.o__Clostridiales.f__Lachnospiraceae.g__Lachnoclostridium | 16.23 |
| 2 | k__Bacteria.p__Chlamydiae.c__Chlamydiia.o__Chlamydiales.f__Chlamydiaceae.g__Chlamydia | 10.58 |
| 3 | k__Bacteria.p__Bacteroidetes.c__Bacteroidia.o__Bacteroidales.f__Bacteroidaceae.g__Bacteroides | 8.98 |
| 4 | k__Bacteria.p__Fibrobacteres.c__Chitinivibrionia.o__Chitinivibrionales.f__Chitinivibrionaceae.g__Chitinivibrio | 8.53 |
| 5 | k__Bacteria.p__Proteobacteria.c__Betaproteobacteria.o__Neisseriales.f__Chromobacteriaceae.g__Gulbenkiania | 8.39 |
| 6 | k__Bacteria.p__Proteobacteria.c__Alphaproteobacteria.o__Rickettsiales.f__Anaplasmataceae.g__Anaplasma | 8.04 |
| 7 | k__Viruses.o__Herpesvirales.f__Herpesviridae.g__Mardivirus | 7.68 |
| 8 | k__Bacteria.p__Proteobacteria.c__Alphaproteobacteria.o__Rhodobacterales.f__Rhodobacteraceae.g__Ruegeria | 7.63 |
| 9 | k__Bacteria.p__Proteobacteria.c__Gammaproteobacteria.o__Enterobacteriales.f__Enterobacteriaceae.g__Proteus | 7.11 |
| 10 | k__Bacteria.p__Actinobacteria.c__Actinobacteria.o__Corynebacteriales.f__Mycobacteriaceae.g__Mycobacterium | 7.07 |
| 11 | k__Bacteria.p__Firmicutes.c__Bacilli.o__Bacillales.f__Paenibacillaceae.g__Paenibacillus | 5.95 |
| 12 | k__Bacteria.p__Proteobacteria.c__Betaproteobacteria.o__Burkholderiales.f__Sutterellaceae.g__Sutterella | 5.60 |
| 13 | k__Archaea.p__Euryarchaeota.c__Halobacteria.o__Halobacteriales.f__Halobacteriaceae.g__Haladaptatus | 5.31 |
| 14 | k__Bacteria.p__Proteobacteria.c__Gammaproteobacteria.o__Aeromonadales.f__Succinivibrionaceae.g__Succinimonas | 5.18 |
| 15 | k__Archaea.p__Euryarchaeota.c__Halobacteria.o__Haloferacales.f__Haloferacaceae.g__Haloquadratum | 4.54 |
| 16 | k__Bacteria.p__Bacteroidetes.c__Cytophagia.o__Cytophagales.f__Flammeovirgaceae.g__Flammeovirga | 4.48 |
| 17 | k__Bacteria.p__Proteobacteria.c__Betaproteobacteria.o__Burkholderiales.g__Tepidimonas | 4.45 |
| 18 | k__Bacteria.p__Proteobacteria.c__Gammaproteobacteria.o__Chromatiales.f__Chromatiaceae.g__Marichromatium | 4.38 |
| 19 | k__Bacteria.p__Firmicutes.c__Bacilli.o__Bacillales.f__Paenibacillaceae.g__Cohnella | 4.18 |
| 20 | k__Bacteria.p__Bacteroidetes.c__Bacteroidia.o__Bacteroidales.f__Porphyromonadaceae.g__Sanguibacteroides | 4.15 |

| **Liver Hepatocellular Carcinoma** | | |
| --- | --- | --- |
| **Ranking** | **Microbial Types** | **Contribution (×10^­-3^)** |
| 1 | k__Bacteria.p__Spirochaetes.c__Spirochaetia.f__Leptospiraceae.g__Leptospira | 41.65 |
| 2 | k__Bacteria.p__Bacteroidetes.c__Flavobacteriia.o__Flavobacteriales.f__Flavobacteriaceae.g__Flavobacterium | 36.16 |
| 3 | k__Bacteria.p__Proteobacteria.c__Gammaproteobacteria.o__Vibrionales.f__Vibrionaceae.g__Grimontia | 35.03 |
| 4 | k__Bacteria.p__Proteobacteria.c__Epsilonproteobacteria.o__Campylobacterales.f__Helicobacteraceae.g__Helicobacter | 28.34 |
| 5 | k__Bacteria.p__Tenericutes.c__Mollicutes.o__Mycoplasmatales.f__Mycoplasmataceae.g__Mycoplasma | 27.48 |
| 6 | k__Bacteria.p__Bacteroidetes.c__Flavobacteriia.o__Flavobacteriales.f__Flavobacteriaceae.g__Chryseobacterium | 24.71 |
| 7 | k__Bacteria.p__Firmicutes.c__Bacilli.o__Bacillales.f__Bacillaceae.g__Fictibacillus | 23.03 |
| 8 | k__Bacteria.p__Proteobacteria.c__Betaproteobacteria.o__Burkholderiales.f__Burkholderiaceae.g__Ralstonia | 20.57 |
| 9 | k__Bacteria.p__Planctomycetes.c__Planctomycetia.o__Planctomycetales.f__Planctomycetaceae.g__Gemmata | 19.00 |
| 10 | k__Bacteria.p__Proteobacteria.c__Betaproteobacteria.o__Burkholderiales.f__Oxalobacteraceae.g__Herbaspirillum | 18.96 |
| 11 | k__Bacteria.p__Firmicutes.c__Clostridia.o__Clostridiales.f__Clostridiaceae.g__Clostridium | 18.47 |
| 12 | k__Bacteria.p__Firmicutes.c__Clostridia.o__Thermoanaerobacterales.f__Thermoanaerobacteraceae.g__Thermoanaerobacter | 18.46 |
| 13 | k__Bacteria.p__Bacteroidetes.c__Bacteroidia.o__Bacteroidales.f__Prevotellaceae.g__Prevotella | 18.26 |
| 14 | k__Archaea.p__Euryarchaeota.c__Methanobacteria.o__Methanobacteriales.f__Methanobacteriaceae.g__Methanobrevibacter | 17.38 |
| 15 | k__Bacteria.p__Proteobacteria.c__Gammaproteobacteria.o__Enterobacteriales.f__Enterobacteriaceae.g__Buchnera | 16.51 |
| 16 | k__Bacteria.p__Thermodesulfobacteria.c__Thermodesulfobacteria.o__Thermodesulfobacteriales.f__Thermodesulfobacteriaceae.g__Thermodesulfobacterium | 16.34 |
| 17 | k__Bacteria.p__Firmicutes.c__Bacilli.o__Bacillales.f__Planococcaceae.g__Paenisporosarcina | 16.21 |
| 18 | k__Bacteria.p__Proteobacteria.c__Gammaproteobacteria.o__Pasteurellales.f__Pasteurellaceae.g__Haemophilus | 15.09 |
| 19 | k__Bacteria.p__Proteobacteria.c__Gammaproteobacteria.o__Enterobacteriales.f__Enterobacteriaceae.g__Pantoea | 14.04 |
| 20 | k__Bacteria.p__Bacteroidetes.c__Flavobacteriia.o__Flavobacteriales.f__Flavobacteriaceae.g__Arenibacter | 13.77 |

| **Lung Adenocarcinoma** | | |
| --- | --- | --- |
| **Ranking** | **Microbial Types** | **Contribution (×10^­-3^)** |
| 1 | k__Bacteria.p__Proteobacteria.c__Gammaproteobacteria.o__Chromatiales.f__Chromatiaceae.g__Marichromatium | 16.35 |
| 2 | k__Bacteria.p__Proteobacteria.c__Gammaproteobacteria.o__Aeromonadales.f__Succinivibrionaceae.g__Succinimonas | 9.60 |
| 3 | k__Bacteria.p__Proteobacteria.c__Gammaproteobacteria.o__Enterobacteriales.f__Enterobacteriaceae.g__Phaseolibacter | 5.87 |
| 4 | k__Bacteria.p__Bacteroidetes.c__Bacteroidia.o__Bacteroidales.f__Porphyromonadaceae.g__Paludibacter | 5.69 |
| 5 | k__Bacteria.p__Cyanobacteria.o__Chroococcales.g__Chamaesiphon | 5.67 |
| 6 | k__Bacteria.p__Acidobacteria.c__Holophagae.o__Holophagales.f__Holophagaceae.g__Holophaga | 5.51 |
| 7 | k__Bacteria.p__Proteobacteria.c__Gammaproteobacteria.o__Vibrionales.f__Vibrionaceae.g__Vibrio | 5.25 |
| 8 | k__Bacteria.p__Proteobacteria.c__Alphaproteobacteria.o__Rhodospirillales.f__Rhodospirillaceae.g__Fodinicurvata | 5.21 |
| 9 | k__Bacteria.p__Proteobacteria.c__Deltaproteobacteria.o__Desulfovibrionales.f__Desulfohalobiaceae.g__Desulfovermiculus | 5.05 |
| 10 | k__Bacteria.p__Proteobacteria.c__Gammaproteobacteria.o__Pseudomonadales.f__Pseudomonadaceae.g__Pseudomonas | 4.87 |
| 11 | k__Bacteria.p__Proteobacteria.c__Betaproteobacteria.o__Burkholderiales.f__Comamonadaceae.g__Comamonas | 4.51 |
| 12 | k__Archaea.p__Euryarchaeota.c__Halobacteria.o__Natrialbales.f__Natrialbaceae.g__Natrialba | 4.41 |
| 13 | k__Bacteria.p__Proteobacteria.c__Alphaproteobacteria.o__Rhodobacterales.f__Rhodobacteraceae.g__Ruegeria | 4.35 |
| 14 | k__Bacteria.p__Planctomycetes.c__Planctomycetia.o__Candidatus_Brocadiales.f__Candidatus_Brocadiaceae.g__Candidatus_Jettenia | 4.31 |
| 15 | k__Bacteria.p__Proteobacteria.c__Gammaproteobacteria.o__Alteromonadales.f__Colwelliaceae.g__Colwellia | 4.27 |
| 16 | k__Bacteria.p__Proteobacteria.c__Gammaproteobacteria.o__Chromatiales.f__Ectothiorhodospiraceae.g__Thioalkalivibrio | 4.12 |
| 17 | k__Bacteria.p__Bacteroidetes.c__Flavobacteriia.o__Flavobacteriales.f__Flavobacteriaceae.g__Lacinutrix | 3.92 |
| 18 | k__Bacteria.p__Verrucomicrobia.c__Opitutae.o__Opitutales.f__Opitutaceae.g__Diplosphaera | 3.86 |
| 19 | k__Bacteria.p__Proteobacteria.c__Gammaproteobacteria.o__Vibrionales.f__Vibrionaceae.g__Enterovibrio | 3.81 |
| 20 | k__Bacteria.p__Firmicutes.c__Clostridia.o__Clostridiales.f__Ruminococcaceae.g__Acetivibrio | 3.63 |

| **Lung Squamous Cell Carcinoma** | | |
| --- | --- | --- |
| **Ranking** | **Microbial Types** | **Contribution (×10^­-3^)** |
| 1 | k__Bacteria.p__Firmicutes.c__Bacilli.o__Bacillales.g__Exiguobacterium | 51.20 |
| 2 | k__Bacteria.p__Proteobacteria.c__Gammaproteobacteria.o__Enterobacteriales.f__Enterobacteriaceae.g__Escherichia | 25.33 |
| 3 | k__Bacteria.p__Firmicutes.c__Bacilli.o__Bacillales.f__Planococcaceae.g__Paenisporosarcina | 24.94 |
| 4 | k__Archaea.p__Euryarchaeota.c__Methanobacteria.o__Methanobacteriales.f__Methanobacteriaceae.g__Methanobrevibacter | 24.35 |
| 5 | k__Bacteria.p__Proteobacteria.c__Gammaproteobacteria.o__Enterobacteriales.f__Enterobacteriaceae.g__Buchnera | 23.50 |
| 6 | k__Bacteria.p__Proteobacteria.c__Gammaproteobacteria.o__Pasteurellales.f__Pasteurellaceae.g__Haemophilus | 21.01 |
| 7 | k__Bacteria.p__Firmicutes.c__Clostridia.o__Thermoanaerobacterales.f__Thermoanaerobacteraceae.g__Caldanaerobacter | 20.48 |
| 8 | k__Bacteria.p__Firmicutes.c__Clostridia.o__Clostridiales.f__Lachnospiraceae.g__Blautia | 19.50 |
| 9 | k__Bacteria.p__Proteobacteria.c__Alphaproteobacteria.o__Rickettsiales.f__Anaplasmataceae.g__Ehrlichia | 18.89 |
| 10 | k__Archaea.p__Euryarchaeota.c__Methanomicrobia.o__Methanosarcinales.f__Methanosarcinaceae.g__Methanosarcina | 17.31 |
| 11 | k__Bacteria.p__Spirochaetes.c__Spirochaetia.o__Spirochaetales.f__Borreliaceae.g__Borrelia | 16.10 |
| 12 | k__Bacteria.p__Proteobacteria.c__Betaproteobacteria.o__Burkholderiales.f__Burkholderiaceae.g__Paraburkholderia | 15.51 |
| 13 | k__Bacteria.p__Planctomycetes.c__Planctomycetia.o__Planctomycetales.f__Planctomycetaceae.g__Gemmata | 14.80 |
| 14 | k__Viruses.o__Tymovirales.f__Betaflexiviridae.g__Carlavirus | 14.28 |
| 15 | k__Bacteria.p__Proteobacteria.c__Acidithiobacillia.o__Acidithiobacillales.f__Acidithiobacillaceae.g__Acidithiobacillus | 14.04 |
| 16 | k__Bacteria.p__Proteobacteria.c__Epsilonproteobacteria.o__Campylobacterales.f__Helicobacteraceae.g__Helicobacter | 12.04 |
| 17 | k__Bacteria.p__Proteobacteria.c__Gammaproteobacteria.o__Enterobacteriales.f__Enterobacteriaceae.g__Citrobacter | 11.19 |
| 18 | k__Bacteria.p__Firmicutes.c__Clostridia.o__Thermoanaerobacterales.f__Thermoanaerobacterales_Family_III._Incertae_Sedis.g__Caldicellulosiruptor | 11.14 |
| 19 | k__Bacteria.p__Bacteroidetes.c__Flavobacteriia.o__Flavobacteriales.f__Flavobacteriaceae.g__Xanthomarina | 10.83 |
| 20 | k__Bacteria.p__Tenericutes.c__Mollicutes.o__Entomoplasmatales.f__Spiroplasmataceae.g__Spiroplasma | 10.31 |

| **Pancreatic Adenocarcinoma** | | |
| --- | --- | --- |
| **Ranking** | **Microbial Types** | **Contribution (×10^­-3^)** |
| 1 | k__Bacteria.p__Bacteroidetes.c__Flavobacteriia.o__Flavobacteriales.f__Flavobacteriaceae.g__Gelidibacter | 10.89 |
| 2 | k__Bacteria.p__Proteobacteria.c__Alphaproteobacteria.o__Rhodobacterales.f__Rhodobacteraceae.g__Tropicibacter | 7.17 |
| 3 | k__Bacteria.p__Proteobacteria.c__Betaproteobacteria.o__Burkholderiales.f__Alcaligenaceae.g__Derxia | 6.48 |
| 4 | k__Archaea.p__Euryarchaeota.c__Halobacteria.o__Halobacteriales.f__Halobacteriaceae.g__Haladaptatus | 6.05 |
| 5 | k__Bacteria.p__Actinobacteria.c__Actinobacteria.o__Pseudonocardiales.f__Pseudonocardiaceae.g__Actinoalloteichus | 5.38 |
| 6 | k__Bacteria.p__Actinobacteria.c__Actinobacteria.o__Actinomycetales.f__Actinomycetaceae.g__Actinomyces | 5.29 |
| 7 | k__Bacteria.p__Proteobacteria.c__Deltaproteobacteria.o__Desulfovibrionales.f__Desulfovibrionaceae.g__Desulfovibrio | 5.05 |
| 8 | k__Bacteria.p__Actinobacteria.c__Actinobacteria.o__Corynebacteriales.f__Nocardiaceae.g__Nocardia | 5.02 |
| 9 | k__Viruses.o__Herpesvirales.f__Herpesviridae.g__Mardivirus | 4.58 |
| 10 | k__Bacteria.p__Proteobacteria.c__Betaproteobacteria.o__Neisseriales.f__Chromobacteriaceae.g__Aquitalea | 4.56 |
| 11 | k__Bacteria.p__Firmicutes.c__Clostridia.o__Clostridiales.f__Ruminococcaceae.g__Ruminiclostridium | 4.55 |
| 12 | k__Bacteria.p__Fibrobacteres.c__Chitinivibrionia.o__Chitinivibrionales.f__Chitinivibrionaceae.g__Chitinivibrio | 4.41 |
| 13 | k__Bacteria.p__Proteobacteria.c__Alphaproteobacteria.o__Sphingomonadales.f__Erythrobacteraceae.g__Altererythrobacter | 4.33 |
| 14 | k__Bacteria.p__Proteobacteria.c__Gammaproteobacteria.o__Alteromonadales.f__Alteromonadaceae.g__Paraglaciecola | 4.25 |
| 15 | k__Archaea.p__Crenarchaeota.c__Thermoprotei.o__Desulfurococcales.f__Desulfurococcaceae.g__Ignicoccus | 4.17 |
| 16 | k__Bacteria.p__Firmicutes.c__Bacilli.o__Bacillales.f__Bacillaceae.g__Virgibacillus | 4.09 |
| 17 | k__Bacteria.p__Actinobacteria.c__Actinobacteria.o__Propionibacteriales.f__Nocardioidaceae.g__Nocardioides | 4.03 |
| 18 | k__Bacteria.p__Bacteroidetes.c__Cytophagia.o__Cytophagales.f__Cytophagaceae.g__Flectobacillus | 4.01 |
| 19 | k__Bacteria.p__Actinobacteria.c__Actinobacteria.o__Pseudonocardiales.f__Pseudonocardiaceae.g__Amycolatopsis | 4.01 |
| 20 | k__Bacteria.p__Proteobacteria.c__Betaproteobacteria.o__Burkholderiales.f__Comamonadaceae.g__Ramlibacter | 3.99 |

| **Rectum Adenocarcinoma** | | |
| --- | --- | --- |
| **Ranking** | **Microbial Types** | **Contribution (×10^­-3^)** |
| 1 | k__Bacteria.p__Proteobacteria.c__Betaproteobacteria.o__Neisseriales.f__Chromobacteriaceae.g__Gulbenkiania | 16.49 |
| 2 | k__Bacteria.p__Proteobacteria.c__Alphaproteobacteria.o__Rhizobiales.f__Brucellaceae.g__Brucella | 16.09 |
| 3 | k__Bacteria.p__Bacteroidetes.c__Bacteroidia.o__Bacteroidales.f__Porphyromonadaceae.g__Sanguibacteroides | 15.57 |
| 4 | k__Bacteria.p__Proteobacteria.c__Deltaproteobacteria.o__Desulfovibrionales.f__Desulfovibrionaceae.g__Bilophila | 14.98 |
| 5 | k__Archaea.p__Euryarchaeota.c__Methanobacteria.o__Methanobacteriales.f__Methanobacteriaceae.g__Methanobacterium | 14.44 |
| 6 | k__Bacteria.p__Proteobacteria.c__Gammaproteobacteria.o__Xanthomonadales.f__Xanthomonadaceae.g__Stenotrophomonas | 14.33 |
| 7 | k__Bacteria.p__Actinobacteria.c__Actinobacteria.o__Corynebacteriales.f__Corynebacteriaceae.g__Corynebacterium | 14.20 |
| 8 | k__Bacteria.p__Proteobacteria.c__Betaproteobacteria.o__Burkholderiales.f__Burkholderiaceae.g__Ralstonia | 14.01 |
| 9 | k__Bacteria.p__Planctomycetes.c__Planctomycetia.o__Candidatus_Brocadiales.f__Candidatus_Brocadiaceae.g__Candidatus_Jettenia | 14.01 |
| 10 | k__Bacteria.p__Firmicutes.c__Tissierellia.o__Tissierellales.f__Peptoniphilaceae.g__Parvimonas | 12.41 |
| 11 | k__Bacteria.p__Proteobacteria.c__Gammaproteobacteria.o__Enterobacteriales.f__Enterobacteriaceae.g__Pantoea | 12.23 |
| 12 | k__Viruses.f__Astroviridae.g__Mamastrovirus | 11.11 |
| 13 | k__Bacteria.p__Spirochaetes.c__Spirochaetia.o__Spirochaetales.f__Spirochaetaceae.g__Treponema | 11.03 |
| 14 | k__Bacteria.p__Proteobacteria.c__Gammaproteobacteria.o__Xanthomonadales.f__Xanthomonadaceae.g__Xylella | 10.98 |
| 15 | k__Bacteria.p__Firmicutes.c__Bacilli.o__Bacillales.f__Planococcaceae.g__Kurthia | 9.70 |
| 16 | k__Bacteria.p__Bacteroidetes.c__Bacteroidia.o__Bacteroidales.f__Prevotellaceae.g__Prevotella | 9.68 |
| 17 | k__Bacteria.p__Actinobacteria.c__Actinobacteria.o__Micrococcales.f__Intrasporangiaceae.g__Tetrasphaera | 9.43 |
| 18 | k__Bacteria.p__Bacteroidetes.c__Flavobacteriia.o__Flavobacteriales.f__Flavobacteriaceae.g__Riemerella | 9.19 |
| 19 | k__Bacteria.p__Firmicutes.c__Bacilli.o__Bacillales.f__Bacillaceae.g__Bacillus | 9.18 |
| 20 | k__Bacteria.p__Proteobacteria.c__Alphaproteobacteria.o__Sphingomonadales.f__Sphingomonadaceae.g__Novosphingobium | 9.10 |

| **Sarcoma** | | |
| --- | --- | --- |
| **Ranking** | **Microbial Types** | **Contribution (×10^­-3^)** |
| 1 | k__Bacteria.p__Firmicutes.c__Clostridia.o__Clostridiales.f__Peptostreptococcaceae.g__Paeniclostridium | 5.42 |
| 2 | k__Viruses.f__Nimaviridae.g__Whispovirus | 5.35 |
| 3 | k__Bacteria.p__Actinobacteria.c__Actinobacteria.o__Geodermatophilales.f__Geodermatophilaceae.g__Geodermatophilus | 5.24 |
| 4 | k__Bacteria.p__Actinobacteria.c__Actinobacteria.o__Micrococcales.f__Brevibacteriaceae.g__Brevibacterium | 4.92 |
| 5 | k__Bacteria.p__Bacteroidetes.c__Cytophagia.o__Cytophagales.f__Flammeovirgaceae.g__Flammeovirga | 4.87 |
| 6 | k__Bacteria.p__Bacteroidetes.c__Cytophagia.o__Cytophagales.f__Cyclobacteriaceae.g__Indibacter | 4.66 |
| 7 | k__Bacteria.p__Bacteroidetes.c__Flavobacteriia.o__Flavobacteriales.f__Flavobacteriaceae.g__Xanthomarina | 4.48 |
| 8 | k__Bacteria.p__Bacteroidetes.c__Flavobacteriia.o__Flavobacteriales.f__Flavobacteriaceae.g__Riemerella | 4.45 |
| 9 | k__Bacteria.p__Proteobacteria.c__Gammaproteobacteria.o__Alteromonadales.f__Alteromonadaceae.g__Marinobacter | 4.24 |
| 10 | k__Bacteria.p__Proteobacteria.c__Gammaproteobacteria.o__Enterobacteriales.f__Enterobacteriaceae.g__Shigella | 4.24 |
| 11 | k__Bacteria.p__Actinobacteria.c__Actinobacteria.o__Micrococcales.f__Dermacoccaceae.g__Kytococcus | 4.22 |
| 12 | k__Bacteria.p__Proteobacteria.c__Gammaproteobacteria.o__Enterobacteriales.f__Enterobacteriaceae.g__Xenorhabdus | 4.20 |
| 13 | k__Bacteria.p__Proteobacteria.c__Betaproteobacteria.o__Burkholderiales.f__Comamonadaceae.g__Acidovorax | 4.19 |
| 14 | k__Bacteria.p__Proteobacteria.c__Alphaproteobacteria.o__Caulobacterales.f__Caulobacteraceae.g__Caulobacter | 4.19 |
| 15 | k__Bacteria.p__Proteobacteria.c__Alphaproteobacteria.o__Rhodobacterales.f__Rhodobacteraceae.g__Tropicibacter | 4.18 |
| 16 | k__Bacteria.p__Actinobacteria.c__Actinobacteria.o__Corynebacteriales.f__Mycobacteriaceae.g__Mycobacterium | 4.18 |
| 17 | k__Bacteria.p__Bacteroidetes.c__Flavobacteriia.o__Flavobacteriales.f__Flavobacteriaceae.g__Olleya | 4.08 |
| 18 | k__Bacteria.p__Cyanobacteria.o__Chroococcales.g__Chamaesiphon | 4.05 |
| 19 | k__Bacteria.p__Firmicutes.c__Bacilli.o__Lactobacillales.f__Carnobacteriaceae.g__Alkalibacterium | 3.98 |
| 20 | k__Bacteria.p__Proteobacteria.c__Alphaproteobacteria.o__Rhizobiales.f__Brucellaceae.g__Ochrobactrum | 3.87 |

| **Skin Cutaneous Melanoma** | | |
| --- | --- | --- |
| **Ranking** | **Microbial Types** | **Contribution (×10^­-3^)** |
| 1 | k__Bacteria.p__Bacteroidetes.c__Bacteroidia.o__Bacteroidales.f__Porphyromonadaceae.g__Sanguibacteroides | 31.77 |
| 2 | k__Bacteria.p__Tenericutes.c__Mollicutes.o__Entomoplasmatales.f__Spiroplasmataceae.g__Spiroplasma | 31.26 |
| 3 | k__Bacteria.p__Firmicutes.c__Clostridia.o__Clostridiales.f__Clostridiaceae.g__Clostridium | 28.73 |
| 4 | k__Bacteria.p__Bacteroidetes.c__Flavobacteriia.o__Flavobacteriales.f__Flavobacteriaceae.g__Chryseobacterium | 23.34 |
| 5 | k__Bacteria.p__Firmicutes.c__Clostridia.o__Thermoanaerobacterales.f__Thermoanaerobacteraceae.g__Caldanaerobacter | 21.22 |
| 6 | k__Bacteria.p__Proteobacteria.c__Gammaproteobacteria.o__Enterobacteriales.f__Enterobacteriaceae.g__Raoultella | 19.53 |
| 7 | k__Bacteria.p__Chlamydiae.c__Chlamydiia.o__Chlamydiales.f__Waddliaceae.g__Waddlia | 18.63 |
| 8 | k__Bacteria.p__Bacteroidetes.c__Cytophagia.o__Cytophagales.f__Cytophagaceae.g__Sporocytophaga | 18.58 |
| 9 | k__Bacteria.p__Firmicutes.c__Bacilli.o__Bacillales.f__Listeriaceae.g__Listeria | 17.96 |
| 10 | k__Bacteria.p__Proteobacteria.c__Betaproteobacteria.o__Burkholderiales.f__Burkholderiaceae.g__Paraburkholderia | 17.33 |
| 11 | k__Bacteria.p__Firmicutes.c__Clostridia.o__Clostridiales.f__Lachnospiraceae.g__Blautia | 16.91 |
| 12 | k__Archaea.p__Euryarchaeota.c__Methanobacteria.o__Methanobacteriales.f__Methanobacteriaceae.g__Methanobrevibacter | 14.97 |
| 13 | k__Bacteria.p__Proteobacteria.c__Alphaproteobacteria.o__Rhodospirillales.f__Acetobacteraceae.g__Acetobacter | 14.85 |
| 14 | k__Bacteria.p__Proteobacteria.c__Deltaproteobacteria.o__Desulfovibrionales.f__Desulfovibrionaceae.g__Lawsonia | 14.58 |
| 15 | k__Bacteria.p__Proteobacteria.c__Epsilonproteobacteria.o__Campylobacterales.f__Helicobacteraceae.g__Helicobacter | 14.31 |
| 16 | k__Bacteria.p__Cyanobacteria.o__Chroococcales.g__Cyanothece | 13.62 |
| 17 | k__Bacteria.p__Proteobacteria.c__Alphaproteobacteria.o__Rhodobacterales.f__Rhodobacteraceae.g__Ruegeria | 13.49 |
| 18 | k__Bacteria.p__Firmicutes.c__Bacilli.o__Bacillales.g__Exiguobacterium | 13.43 |
| 19 | k__Bacteria.p__Spirochaetes.c__Spirochaetia.o__Spirochaetales.f__Borreliaceae.g__Borrelia | 12.32 |
| 20 | k__Bacteria.p__Spirochaetes.c__Spirochaetia.f__Leptospiraceae.g__Leptospira | 12.17 |

| **Thymoma** | | |
| --- | --- | --- |
| **Ranking** | **Microbial Types** | **Contribution (×10^­-3^)** |
| 1 | k__Bacteria.p__Proteobacteria.c__Gammaproteobacteria.o__Legionellales.f__Legionellaceae.g__Legionella | 10.90 |
| 2 | k__Bacteria.p__Firmicutes.c__Clostridia.o__Clostridiales.f__Lachnospiraceae.g__Lachnoclostridium | 10.01 |
| 3 | k__Bacteria.p__Proteobacteria.c__Gammaproteobacteria.o__Alteromonadales.f__Pseudoalteromonadaceae.g__Algicola | 7.73 |
| 4 | k__Bacteria.p__Proteobacteria.c__Alphaproteobacteria.o__Rhodospirillales.f__Acetobacteraceae.g__Gluconacetobacter | 7.35 |
| 5 | k__Bacteria.p__Proteobacteria.c__Gammaproteobacteria.o__Enterobacteriales.f__Enterobacteriaceae.g__Proteus | 7.01 |
| 6 | k__Bacteria.p__Proteobacteria.c__Betaproteobacteria.o__Burkholderiales.f__Comamonadaceae.g__Comamonas | 6.50 |
| 7 | k__Viruses.o__Caudovirales.f__Myoviridae.g__Spo1virus | 6.14 |
| 8 | k__Bacteria.p__Proteobacteria.c__Gammaproteobacteria.o__Chromatiales.f__Ectothiorhodospiraceae.g__Thiorhodospira | 5.93 |
| 9 | k__Viruses.f__Papillomaviridae.g__Alphapapillomavirus | 5.52 |
| 10 | k__Bacteria.p__Proteobacteria.c__Alphaproteobacteria.o__Rhodospirillales.f__Acetobacteraceae.g__Acidiphilium | 5.39 |
| 11 | k__Bacteria.p__Proteobacteria.c__Gammaproteobacteria.o__Vibrionales.f__Vibrionaceae.g__Enterovibrio | 5.23 |
| 12 | k__Bacteria.p__Proteobacteria.c__Gammaproteobacteria.o__Pseudomonadales.f__Moraxellaceae.g__Moraxella | 5.19 |
| 13 | k__Bacteria.p__Bacteroidetes.c__Flavobacteriia.o__Flavobacteriales.f__Flavobacteriaceae.g__Kordia | 4.80 |
| 14 | k__Bacteria.p__Cyanobacteria.o__Oscillatoriales.g__Spirulina | 4.80 |
| 15 | k__Bacteria.p__Bacteroidetes.c__Bacteroidia.o__Bacteroidales.f__Prevotellaceae.g__Paraprevotella | 4.70 |
| 16 | k__Bacteria.p__Nitrospirae.c__Nitrospira.o__Nitrospirales.f__Nitrospiraceae.g__Nitrospira | 4.70 |
| 17 | k__Bacteria.p__Actinobacteria.c__Actinobacteria.o__Micrococcales.f__Micrococcaceae.g__Arthrobacter | 4.65 |
| 18 | k__Bacteria.p__Proteobacteria.c__Alphaproteobacteria.o__Caulobacterales.f__Caulobacteraceae.g__Phenylobacterium | 4.43 |
| 19 | k__Bacteria.p__Proteobacteria.c__Gammaproteobacteria.o__Enterobacteriales.f__Enterobacteriaceae.g__Edwardsiella | 4.35 |
| 20 | k__Viruses.f__Polydnaviridae.g__Ichnovirus | 4.29 |

| **Uterine Corpus Endometrial Carcinoma** | | |
| --- | --- | --- |
| **Ranking** | **Microbial Types** | **Contribution (×10^­-3^)** |
| 1 | k__Bacteria.p__Actinobacteria.c__Actinobacteria.o__Micrococcales.f__Microbacteriaceae.g__Plantibacter | 10.32 |
| 2 | k__Viruses.f__Nimaviridae.g__Whispovirus | 9.36 |
| 3 | k__Bacteria.p__Proteobacteria.c__Alphaproteobacteria.o__Rhizobiales.f__Aurantimonadaceae.g__Aureimonas | 6.87 |
| 4 | k__Bacteria.p__Proteobacteria.c__Alphaproteobacteria.o__Rhodobacterales.f__Rhodobacteraceae.g__Pseudorhodobacter | 6.56 |
| 5 | k__Bacteria.p__Firmicutes.c__Bacilli.o__Bacillales.g__Acidibacillus | 6.20 |
| 6 | k__Bacteria.p__Proteobacteria.c__Betaproteobacteria.o__Rhodocyclales.f__Rhodocyclaceae.g__Dechloromonas | 5.77 |
| 7 | k__Bacteria.p__Proteobacteria.c__Betaproteobacteria.o__Burkholderiales.f__Comamonadaceae.g__Ramlibacter | 5.75 |
| 8 | k__Bacteria.p__Firmicutes.c__Clostridia.o__Clostridiales.f__Peptostreptococcaceae.g__Paeniclostridium | 5.45 |
| 9 | k__Bacteria.p__Bacteroidetes.c__Sphingobacteriia.o__Sphingobacteriales.f__Sphingobacteriaceae.g__Mucilaginibacter | 5.24 |
| 10 | k__Bacteria.p__Proteobacteria.c__Gammaproteobacteria.o__Enterobacteriales.f__Enterobacteriaceae.g__Erwinia | 4.86 |
| 11 | k__Bacteria.p__Proteobacteria.c__Alphaproteobacteria.o__Rhodospirillales.f__Rhodospirillaceae.g__Azospirillum | 4.63 |
| 12 | k__Bacteria.p__Actinobacteria.c__Actinobacteria.o__Micrococcales.f__Cellulomonadaceae.g__Cellulomonas | 4.30 |
| 13 | k__Bacteria.p__Proteobacteria.c__Gammaproteobacteria.o__Enterobacteriales.f__Enterobacteriaceae.g__Pantoea | 4.11 |
| 14 | k__Bacteria.p__Proteobacteria.c__Alphaproteobacteria.o__Rhizobiales.f__Bradyrhizobiaceae.g__Nitrobacter | 4.04 |
| 15 | k__Bacteria.p__Proteobacteria.c__Gammaproteobacteria.o__Enterobacteriales.f__Enterobacteriaceae.g__Enterobacter | 4.04 |
| 16 | k__Bacteria.p__Actinobacteria.c__Actinobacteria.o__Micrococcales.f__Demequinaceae.g__Lysinimicrobium | 4.00 |
| 17 | k__Bacteria.p__Proteobacteria.c__Betaproteobacteria.o__Rhodocyclales.f__Rhodocyclaceae.g__Thauera | 3.99 |
| 18 | k__Bacteria.p__Proteobacteria.c__Alphaproteobacteria.o__Rhizobiales.f__Bradyrhizobiaceae.g__Rhodopseudomonas | 3.99 |
| 19 | k__Bacteria.p__Proteobacteria.c__Alphaproteobacteria.o__Rhizobiales.f__Hyphomicrobiaceae.g__Hyphomicrobium | 3.98 |
| 20 | k__Bacteria.p__Proteobacteria.c__Alphaproteobacteria.o__Rhodospirillales.f__Acetobacteraceae.g__Saccharibacter | 3.93 |

| **Uveal Melanoma** | | |
| --- | --- | --- |
| **Ranking** | **Microbial Types** | **Contribution (×10^­-3^)** |
| 1 | k__Bacteria.p__Proteobacteria.c__Gammaproteobacteria.o__Enterobacteriales.f__Enterobacteriaceae.g__Lelliottia | 16.10 |
| 2 | k__Bacteria.p__Proteobacteria.c__Betaproteobacteria.o__Burkholderiales.f__Comamonadaceae.g__Limnohabitans | 12.57 |
| 3 | k__Bacteria.p__Proteobacteria.c__Alphaproteobacteria.o__Rhizobiales.f__Rhizobiaceae.g__Ensifer | 9.89 |
| 4 | k__Bacteria.p__Proteobacteria.c__Gammaproteobacteria.o__Alteromonadales.f__Pseudoalteromonadaceae.g__Algicola | 9.73 |
| 5 | k__Bacteria.p__Actinobacteria.c__Actinobacteria.o__Micrococcales.f__Micrococcaceae.g__Nesterenkonia | 9.25 |
| 6 | k__Bacteria.p__Actinobacteria.c__Actinobacteria.o__Micrococcales.f__Microbacteriaceae.g__Frondihabitans | 8.42 |
| 7 | k__Bacteria.p__Proteobacteria.c__Gammaproteobacteria.o__Methylococcales.f__Methylococcaceae.g__Methylobacter | 7.63 |
| 8 | k__Bacteria.p__Proteobacteria.c__Gammaproteobacteria.o__Pseudomonadales.f__Moraxellaceae.g__Psychrobacter | 7.15 |
| 9 | k__Bacteria.p__Actinobacteria.c__Actinobacteria.o__Pseudonocardiales.f__Pseudonocardiaceae.g__Alloactinosynnema | 6.94 |
| 10 | k__Bacteria.p__Acidobacteria.c__Holophagae.o__Holophagales.f__Holophagaceae.g__Holophaga | 6.74 |
| 11 | k__Bacteria.p__Proteobacteria.c__Betaproteobacteria.o__Burkholderiales.f__Comamonadaceae.g__Delftia | 6.68 |
| 12 | k__Bacteria.p__Actinobacteria.c__Actinobacteria.o__Pseudonocardiales.f__Pseudonocardiaceae.g__Saccharopolyspora | 6.52 |
| 13 | k__Bacteria.p__Proteobacteria.c__Gammaproteobacteria.o__Pasteurellales.f__Pasteurellaceae.g__Actinobacillus | 6.42 |
| 14 | k__Bacteria.p__Firmicutes.c__Tissierellia.o__Tissierellales.f__Peptoniphilaceae.g__Peptoniphilus | 6.28 |
| 15 | k__Bacteria.p__Proteobacteria.c__Betaproteobacteria.o__Burkholderiales.f__Alcaligenaceae.g__Achromobacter | 6.16 |
| 16 | k__Bacteria.p__Actinobacteria.c__Actinobacteria.o__Micrococcales.f__Microbacteriaceae.g__Pseudoclavibacter | 6.16 |
| 17 | k__Bacteria.p__Firmicutes.c__Bacilli.o__Bacillales.f__Bacillaceae.g__Domibacillus | 6.12 |
| 18 | k__Archaea.p__Euryarchaeota.c__Halobacteria.o__Natrialbales.f__Natrialbaceae.g__Halovivax | 5.80 |
| 19 | k__Bacteria.p__Proteobacteria.c__Alphaproteobacteria.o__Rhodobacterales.f__Rhodobacteraceae.g__Tropicibacter | 5.74 |
| 20 | k__Archaea.p__Euryarchaeota.c__Halobacteria.o__Haloferacales.f__Haloferacaceae.g__Halolamina | 5.24 |

**Supplementary Table 3. Top 20 outstanding host gene with highest contribution of each cancer in subtype prediction.**

| **Adrenocortical Carcinoma** | | |
| --- | --- | --- |
| **Ranking** | **Host Genes** | **Contribution (×10^­-3^)** |
| 1 | LHX4-AS1 | 19.47 |
| 2 | SLC29A4 | 16.35 |
| 3 | COPS7B | 15.84 |
| 4 | RMI2 | 13.71 |
| 5 | FSCN1 | 11.78 |
| 6 | GATAD2B | 11.24 |
| 7 | PRCC | 9.23 |
| 8 | DMKN | 8.31 |
| 9 | GPR137C | 8.30 |
| 10 | OTX1 | 8.22 |
| 11 | ENTPD8 | 8.10 |
| 12 | HAUS8 | 8.08 |
| 13 | KPNB1 | 7.99 |
| 14 | FARP1 | 7.68 |
| 15 | FAM83H | 7.62 |
| 16 | CCNB1IP1 | 7.62 |
| 17 | CENPA | 7.58 |
| 18 | HDAC4 | 7.37 |
| 19 | SGPP2 | 7.14 |
| 20 | INTS7 | 6.97 |

| **Bladder Urothelial Carcinoma** | | |
| --- | --- | --- |
| **Ranking** | **Host Genes** | **Contribution (×10^­-3^)** |
| 1 | TOX3 | 15.73 |
| 2 | CHST15 | 14.31 |
| 3 | PPFIBP2 | 9.72 |
| 4 | MTHFD1L | 9.21 |
| 5 | ZBTB7C | 8.74 |
| 6 | FAAH | 8.33 |
| 7 | ENGASE | 8.32 |
| 8 | CYB5A | 7.80 |
| 9 | S100A5 | 7.71 |
| 10 | SPHK1 | 7.46 |
| 11 | VSIG2 | 7.33 |
| 12 | HS3ST3B1 | 7.33 |
| 13 | PDCD1LG2 | 6.96 |
| 14 | CORO1C | 6.70 |
| 15 | BHMT | 6.32 |
| 16 | MAP7D1 | 6.14 |
| 17 | MCF2L | 6.11 |
| 18 | OVGP1 | 5.70 |
| 19 | CCDC64B | 5.70 |
| 20 | OSBPL6 | 5.66 |

| **Breast Invasive Carcinoma** | | |
| --- | --- | --- |
| **Ranking** | **Host Genes** | **Contribution (×10^­-3^)** |
| 1 | TNFRSF14 | 15.10 |
| 2 | CROCC | 9.88 |
| 3 | PPP1R12C | 9.47 |
| 4 | SSBP4 | 9.04 |
| 5 | XPOT | 8.53 |
| 6 | TMEM240 | 8.11 |
| 7 | CCDC94 | 8.01 |
| 8 | TNFSF12 | 6.79 |
| 9 | TNFRSF4 | 6.40 |
| 10 | FYTTD1 | 6.19 |
| 11 | INAFM1 | 6.15 |
| 12 | WDR13 | 5.71 |
| 13 | IMPAD1 | 5.55 |
| 14 | MBD3 | 5.49 |
| 15 | PDLIM2 | 5.43 |
| 16 | GPR35 | 5.40 |
| 17 | ATP6V1A | 5.33 |
| 18 | BGLAP | 5.10 |
| 19 | FBXL15 | 4.78 |
| 20 | ZNF358 | 4.74 |

| **Cervical Squamous Cell Carcinoma and Endocervical Adenocarcinoma** | | |
| --- | --- | --- |
| **Ranking** | **Host Genes** | **Contribution (×10^­-3^)** |
| 1 | SNRPG | 3.12 |
| 2 | ZNF518A | 3.07 |
| 3 | KRT10 | 2.75 |
| 4 | UQCRH | 2.50 |
| 5 | KAL1 | 2.40 |
| 6 | PXK | 2.38 |
| 7 | DAPK3 | 2.21 |
| 8 | ZFPM1 | 2.19 |
| 9 | SYNPO | 2.08 |
| 10 | MRPS12 | 1.99 |
| 11 | RNF20 | 1.94 |
| 12 | RSPH4A | 1.85 |
| 13 | SETDB2 | 1.78 |
| 14 | RPL13A | 1.78 |
| 15 | OAZ3 | 1.66 |
| 16 | SURF2 | 1.66 |
| 17 | HES7 | 1.65 |
| 18 | TGFBR1 | 1.65 |
| 19 | AKAP11 | 1.58 |
| 20 | UBE4A | 1.54 |

| **Colon Adenocarcinoma** | | |
| --- | --- | --- |
| **Ranking** | **Host Genes** | **Contribution (×10^­-3^)** |
| 1 | TFB2M | 7.27 |
| 2 | TMEM91 | 7.19 |
| 3 | ANKS3 | 6.76 |
| 4 | ZMPSTE24 | 5.82 |
| 5 | MAD2L1 | 5.81 |
| 6 | SLC25A24 | 5.64 |
| 7 | FAR2 | 5.57 |
| 8 | DTWD2 | 5.40 |
| 9 | NISCH | 5.30 |
| 10 | BAI1 | 5.04 |
| 11 | ENDOV | 4.65 |
| 12 | TPMT | 4.56 |
| 13 | TM9SF3 | 4.53 |
| 14 | HDAC10 | 4.48 |
| 15 | RARS | 4.47 |
| 16 | GLCE | 4.31 |
| 17 | DSCR3 | 4.24 |
| 18 | ADD3 | 4.22 |
| 19 | KLC1 | 4.11 |
| 20 | LZIC | 4.05 |

| **Head and Neck Squamous Cell Carcinoma** | | |
| --- | --- | --- |
| **Ranking** | **Host Genes** | **Contribution (×10^­-3^)** |
| 1 | MAGIX | 6.75 |
| 2 | SPACA4 | 5.98 |
| 3 | PLCXD1 | 5.63 |
| 4 | LLGL2 | 5.41 |
| 5 | P4HA2 | 5.34 |
| 6 | PRSS36 | 5.13 |
| 7 | SPOCK1 | 4.70 |
| 8 | ITGA5 | 4.42 |
| 9 | ROS1 | 4.41 |
| 10 | ANXA9 | 4.41 |
| 11 | ATXN7L2 | 4.10 |
| 12 | MRPS25 | 4.04 |
| 13 | CREG2 | 3.72 |
| 14 | ACTN1 | 3.68 |
| 15 | GDPD3 | 3.65 |
| 16 | CHDH | 3.34 |
| 17 | CLIC3 | 3.23 |
| 18 | ACAA1 | 3.17 |
| 19 | ATG9B | 3.16 |
| 20 | PLAU | 3.14 |

| **Kidney Chromophobe** | | |
| --- | --- | --- |
| **Ranking** | **Host Genes** | **Contribution (×10^­-3^)** |
| 1 | SMAD5 | 18.52 |
| 2 | DMXL1 | 17.75 |
| 3 | CEP128 | 15.85 |
| 4 | HEATR1 | 12.72 |
| 5 | EIF2S3 | 10.31 |
| 6 | BPTF | 10.21 |
| 7 | NEMF | 9.33 |
| 8 | CHM | 9.31 |
| 9 | C12orf4 | 9.31 |
| 10 | CAND1 | 8.84 |
| 11 | YIPF5 | 8.82 |
| 12 | PHC3 | 8.81 |
| 13 | HSPA8 | 8.49 |
| 14 | KIAA1143 | 8.37 |
| 15 | TBK1 | 8.28 |
| 16 | FAM179B | 8.27 |
| 17 | DEK | 8.26 |
| 18 | STAG1 | 8.26 |
| 19 | POLR2B | 8.20 |
| 20 | DDX46 | 8.19 |

| **Kidney Renal Clear Cell Carcinoma** | | |
| --- | --- | --- |
| **Ranking** | **Host Genes** | **Contribution (×10^­-3^)** |
| 1 | TAF10 | 17.38 |
| 2 | TSPYL1 | 13.44 |
| 3 | PRKAA2 | 10.96 |
| 4 | FRMD3 | 10.49 |
| 5 | FNBP1L | 9.87 |
| 6 | TMEM245 | 9.75 |
| 7 | NR3C2 | 8.68 |
| 8 | WDR72 | 8.61 |
| 9 | KIDINS220 | 8.20 |
| 10 | AQR | 8.09 |
| 11 | MEGF9 | 8.05 |
| 12 | SUCLA2 | 8.05 |
| 13 | BHLHB9 | 8.02 |
| 14 | MYO9A | 7.81 |
| 15 | MUT | 7.53 |
| 16 | KIF1B | 6.99 |
| 17 | DTX2 | 6.97 |
| 18 | PCCA | 6.79 |
| 19 | CDC14B | 6.62 |
| 20 | PINK1 | 6.44 |

| **Kidney Renal Papillary Cell Carcinoma** | | |
| --- | --- | --- |
| **Ranking** | **Host Genes** | **Contribution (×10^­-3^)** |
| 1 | RAB27A | 19.32 |
| 2 | TSPAN5 | 15.40 |
| 3 | ECI1 | 14.02 |
| 4 | ITGA5 | 12.52 |
| 5 | ATPAF2 | 11.98 |
| 6 | OCIAD2 | 11.64 |
| 7 | GLYAT | 9.33 |
| 8 | RASD2 | 8.71 |
| 9 | CUL4B | 8.66 |
| 10 | CCDC106 | 8.37 |
| 11 | C3orf70 | 8.27 |
| 12 | SLC7A2 | 8.19 |
| 13 | PARP1 | 8.18 |
| 14 | LAMP3 | 7.66 |
| 15 | TEX264 | 7.59 |
| 16 | ASAP1 | 7.09 |
| 17 | LIMS1 | 7.07 |
| 18 | PLS3 | 6.58 |
| 19 | TPM4 | 6.48 |
| 20 | LRRC8C | 6.46 |

| **Brain Lower Grade Glioma** | | |
| --- | --- | --- |
| **Ranking** | **Host Genes** | **Contribution (×10^­-3^)** |
| 1 | ADAM12 | 18.19 |
| 2 | LAMC1 | 18.00 |
| 3 | FKBP9 | 14.81 |
| 4 | FAM114A1 | 13.26 |
| 5 | THRA | 12.34 |
| 6 | GGH | 12.26 |
| 7 | PHYHIPL | 12.06 |
| 8 | DCTD | 11.90 |
| 9 | E2F7 | 11.61 |
| 10 | LATS2 | 11.58 |
| 11 | PCBP3 | 11.31 |
| 12 | MRC2 | 10.51 |
| 13 | FAM109B | 9.22 |
| 14 | METTL7B | 8.88 |
| 15 | GHITM | 8.03 |
| 16 | MEOX2 | 7.89 |
| 17 | ARL3 | 7.69 |
| 18 | IGFBP2 | 7.53 |
| 19 | CD58 | 7.45 |
| 20 | CDK2 | 7.32 |

| **Liver Hepatocellular Carcinoma** | | |
| --- | --- | --- |
| **Ranking** | **Host Genes** | **Contribution (×10^­-3^)** |
| 1 | VGF | 3.64 |
| 2 | GTF2H3 | 2.81 |
| 3 | ZNF581 | 2.31 |
| 4 | GNB2L1 | 2.07 |
| 5 | LPHN2 | 2.05 |
| 6 | NDUFA1 | 2.04 |
| 7 | EIF5 | 1.76 |
| 8 | NANOS3 | 1.73 |
| 9 | MED19 | 1.61 |
| 10 | SRP72 | 1.58 |
| 11 | CCZ1 | 1.55 |
| 12 | ATP8A2 | 1.51 |
| 13 | TIMM10 | 1.47 |
| 14 | RPS21 | 1.46 |
| 15 | RPS14 | 1.41 |
| 16 | RPS16 | 1.41 |
| 17 | CRMP1 | 1.40 |
| 18 | MAN2C1 | 1.39 |
| 19 | CES5A | 1.34 |
| 20 | PRH2 | 1.33 |

| **Lung Adenocarcinoma** | | |
| --- | --- | --- |
| **Ranking** | **Host Genes** | **Contribution (×10^­-3^)** |
| 1 | RHOD | 8.04 |
| 2 | TP53INP1 | 7.58 |
| 3 | CTB-133G6.1 | 7.56 |
| 4 | AGO4 | 7.21 |
| 5 | STX16-NPEPL1 | 7.11 |
| 6 | SLC26A5 | 6.94 |
| 7 | ANKRD44 | 6.36 |
| 8 | FCHSD2 | 6.24 |
| 9 | ATP8A1 | 6.05 |
| 10 | BZRAP1 | 5.84 |
| 11 | TRMT10B | 5.75 |
| 12 | CLK4 | 5.68 |
| 13 | SLC2A1 | 5.62 |
| 14 | SEC31B | 5.35 |
| 15 | BTG2 | 5.16 |
| 16 | ZNF641 | 4.90 |
| 17 | PPFIBP2 | 4.79 |
| 18 | PLCB2 | 4.71 |
| 19 | DNAH1 | 4.71 |
| 20 | FUCA2 | 4.51 |

| **Lung Squamous Cell Carcinoma** | | |
| --- | --- | --- |
| **Ranking** | **Host Genes** | **Contribution (×10^­-3^)** |
| 1 | TCERG1 | 5.08 |
| 2 | RBM6 | 4.17 |
| 3 | BTAF1 | 3.88 |
| 4 | MTRNR2L12 | 3.39 |
| 5 | ACIN1 | 3.29 |
| 6 | NKTR | 3.27 |
| 7 | PRSS53 | 3.27 |
| 8 | PRRT2 | 3.02 |
| 9 | TAF1C | 3.00 |
| 10 | DDX17 | 2.91 |
| 11 | RNPC3 | 2.78 |
| 12 | SLC28A2 | 2.74 |
| 13 | MYSM1 | 2.48 |
| 14 | CLPB | 2.47 |
| 15 | TAS2R20 | 2.45 |
| 16 | PRPF38B | 2.40 |
| 17 | AL158801.1 | 2.35 |
| 18 | C22orf46 | 2.34 |
| 19 | AMY2B | 2.31 |
| 20 | ZFC3H1 | 2.29 |

| **Pancreatic Adenocarcinoma** | | |
| --- | --- | --- |
| **Ranking** | **Host Genes** | **Contribution (×10^­-3^)** |
| 1 | ZNF781 | 26.72 |
| 2 | RAB25 | 22.30 |
| 3 | GNG2 | 21.26 |
| 4 | OAZ2 | 14.47 |
| 5 | CXorf36 | 14.11 |
| 6 | NOVA1 | 12.98 |
| 7 | ANK2 | 12.12 |
| 8 | GNG7 | 11.16 |
| 9 | ZDHHC15 | 10.29 |
| 10 | FAM83H | 9.48 |
| 11 | ZNF667 | 9.25 |
| 12 | TSPAN7 | 9.08 |
| 13 | CERKL | 8.59 |
| 14 | GPR162 | 8.29 |
| 15 | CORO2B | 7.98 |
| 16 | GIMAP1 | 7.91 |
| 17 | PALD1 | 7.85 |
| 18 | ISCA1 | 7.80 |
| 19 | SESN1 | 7.57 |
| 20 | GIMAP4 | 7.54 |

| **Rectum Adenocarcinoma** | | |
| --- | --- | --- |
| **Ranking** | **Host Genes** | **Contribution (×10^­-3^)** |
| 1 | MARK1 | 6.39 |
| 2 | SOCS5 | 6.26 |
| 3 | SYCP2 | 5.53 |
| 4 | JMJD1C | 4.59 |
| 5 | ZNF296 | 4.43 |
| 6 | XPNPEP3 | 4.06 |
| 7 | RNF38 | 4.01 |
| 8 | SNAPC5 | 4.01 |
| 9 | CRIM1 | 3.95 |
| 10 | STAM2 | 3.59 |
| 11 | TRAPPC5 | 3.53 |
| 12 | CCDC186 | 3.46 |
| 13 | TMEM106B | 3.38 |
| 14 | CMTM7 | 3.32 |
| 15 | THAP6 | 3.30 |
| 16 | CHTF8 | 3.28 |
| 17 | DUS2 | 3.17 |
| 18 | ZNF580 | 3.15 |
| 19 | DPM3 | 3.15 |
| 20 | AP1S1 | 3.15 |

| **Sarcoma** | | |
| --- | --- | --- |
| **Ranking** | **Host Genes** | **Contribution (×10^­-3^)** |
| 1 | HMGCR | 10.56 |
| 2 | FBXW8 | 9.15 |
| 3 | TMEM176B | 8.50 |
| 4 | TCF3 | 7.09 |
| 5 | VAMP5 | 6.94 |
| 6 | EPHB4 | 6.73 |
| 7 | DNA2 | 6.70 |
| 8 | SNX20 | 6.60 |
| 9 | SYNGR2 | 6.41 |
| 10 | CYSTM1 | 6.28 |
| 11 | NCKAP1L | 6.05 |
| 12 | G2E3 | 6.01 |
| 13 | PPIL4 | 5.98 |
| 14 | C16orf54 | 5.89 |
| 15 | NMRK1 | 5.51 |
| 16 | ZBED4 | 5.49 |
| 17 | B3GALNT1 | 5.44 |
| 18 | PSTPIP1 | 5.40 |
| 19 | C10orf128 | 5.21 |
| 20 | MPHOSPH9 | 5.07 |

| **Skin Cutaneous Melanoma** | | |
| --- | --- | --- |
| **Ranking** | **Host Genes** | **Contribution (×10^­-3^)** |
| 1 | LMNTD2 | 6.55 |
| 2 | CYP51A1 | 6.40 |
| 3 | CSDE1 | 5.13 |
| 4 | ROMO1 | 5.06 |
| 5 | SEPP1 | 4.75 |
| 6 | TMEM160 | 4.54 |
| 7 | GOLGA1 | 4.32 |
| 8 | CLASRP | 4.23 |
| 9 | TPGS1 | 4.21 |
| 10 | AHCYL1 | 4.02 |
| 11 | CCS | 3.98 |
| 12 | TANC1 | 3.90 |
| 13 | COL8A2 | 3.82 |
| 14 | OGFR | 3.82 |
| 15 | DOHH | 3.80 |
| 16 | SRP72 | 3.74 |
| 17 | METTL8 | 3.73 |
| 18 | BOLA2B | 3.72 |
| 19 | C17orf89 | 3.72 |
| 20 | NNT | 3.72 |

| **Thymoma** | | |
| --- | --- | --- |
| **Ranking** | **Host Genes** | **Contribution (×10^­-3^)** |
| 1 | RAB13 | 17.37 |
| 2 | STAG3 | 12.88 |
| 3 | BUD13 | 12.17 |
| 4 | GSTK1 | 12.08 |
| 5 | PPIP5K1 | 10.92 |
| 6 | COQ2 | 10.45 |
| 7 | FAHD2A | 9.67 |
| 8 | ST7 | 8.98 |
| 9 | YPEL1 | 8.89 |
| 10 | ETV7 | 7.37 |
| 11 | FBXO6 | 6.91 |
| 12 | MAPRE3 | 6.83 |
| 13 | H1FX | 6.50 |
| 14 | GABPB1 | 6.40 |
| 15 | C22orf15 | 6.27 |
| 16 | TFDP2 | 6.21 |
| 17 | MYH14 | 6.05 |
| 18 | TRIM69 | 6.05 |
| 19 | TBC1D1 | 6.02 |
| 20 | TSHR | 5.94 |

| **Uterine Corpus Endometrial Carcinoma** | | |
| --- | --- | --- |
| **Ranking** | **Host Genes** | **Contribution (×10^­-3^)** |
| 1 | ANKFN1 | 13.60 |
| 2 | SPDEF | 12.54 |
| 3 | RFC4 | 12.26 |
| 4 | MYT1 | 10.35 |
| 5 | KIAA1324 | 9.64 |
| 6 | KCNK9 | 9.07 |
| 7 | TFF3 | 8.93 |
| 8 | SLC5A1 | 8.89 |
| 9 | CDKN2A | 8.57 |
| 10 | NPDC1 | 8.36 |
| 11 | UCHL1 | 8.08 |
| 12 | IL20RA | 7.85 |
| 13 | TPX2 | 7.78 |
| 14 | DLL3 | 7.63 |
| 15 | VMAC | 6.91 |
| 16 | NANS | 6.72 |
| 17 | IGF2BP1 | 6.39 |
| 18 | CEACAM1 | 6.33 |
| 19 | CREB3L1 | 6.24 |
| 20 | TUBB4A | 6.19 |

| **Uveal Melanoma** | | |
| --- | --- | --- |
| **Ranking** | **Host Genes** | **Contribution (×10^­-3^)** |
| 1 | SAP30 | 24.65 |
| 2 | COL9A3 | 18.99 |
| 3 | FBXO17 | 17.56 |
| 4 | SGSM2 | 17.56 |
| 5 | EIF4E2 | 17.51 |
| 6 | COQ6 | 17.08 |
| 7 | AZGP1 | 16.67 |
| 8 | VOPP1 | 16.63 |
| 9 | SLC25A38 | 12.82 |
| 10 | ACKR2 | 12.14 |
| 11 | PPP1R14C | 10.84 |
| 12 | MGST2 | 9.49 |
| 13 | HTRA3 | 9.49 |
| 14 | DUSP14 | 9.01 |
| 15 | P2RX6 | 9.01 |
| 16 | SOCS2 | 9.00 |
| 17 | HTR2B | 9.00 |
| 18 | VAX1 | 9.00 |
| 19 | ADAM11 | 8.56 |
| 20 | NRROS | 8.55 |
